# Screening for *Trypanosoma cruzi* infection in immigrants and refugees: Systematic review and recommendations from the Spanish Society of Infectious Diseases and Clinical Microbiology 

**DOI:** 10.2807/1560-7917.ES.2020.25.8.1900393

**Published:** 2020-02-27

**Authors:** María Velasco, Luis Andrés Gimeno-Feliú, Israel Molina, Joaquín Salas-Coronas, Ivan Solà, Begoña Monge-Maillo, Diego Torrús-Tendero, Joan Caylà, Ena Niño de Guzmán, JL Pérez Arellano, Jose A Pérez-Molina

**Affiliations:** 1 Infectious and Tropical Medicine Section, Internal Medicine Department, Hospital Universitario Fundación Alcorcón, Madrid, Spain; 2 San Pablo Health Centre, Zaragoza, Spain; 3 Department of Medicine, Psychiatry and Dermatology, University of Zaragoza. EpiChron Research Group on Chronic Diseases, Aragón Health Sciences Institute (IACS), IIS Aragón, Hospital Universitario Miguel Servet, Zaragoza, Spain; 4 Health Services Research on Chronic Patients Network (REDISSEC), Instituto de Salud Carlos III, Madrid, Spain; 5 Infectious Diseases Department, Hospital Universitario Vall d'Hebron, PROSICS Barcelona, Universitat Autònoma de Barcelona, Barcelona, Spain; 6 Tropical Medicine Unit. Hospital de Poniente, El Ejido, Almería, Spain; 7 Iberoamerican Cochrane Centre, Biomedical Research Institute Sant Pau (IIB Sant Pau), Barcelona, Spain; 8 CIBER of Epidemiology and Public Health (CIBERESP), Barcelona, Spain; 9 National Referral Centre for Tropical Diseases, Infectious Diseases Department, Hospital Universitario Ramón y Cajal, IRYCIS, Madrid, Spain; 10 Referral Unit for Imported Infections and International Health. Infectious Diseases Unit, Hospital General Universitario de Alicante. Parasitology Area, Miguel Hernández University, Alicante, Spain; 11 Fundació de la Unitat d'Investigació en Tuberculosis (FuiTB), TB Research Unit, Barcelona, Spain; 12 Infectious Diseases and Tropical Medicine Unit, Hospital Insular de Las Palmas. Las Palmas de Gran Canaria, Spain; 13 Medical and Surgical Sciences Department. Universidad de Las Palmas de Gran Canaria, Las Palmas de Gran Canaria, Spain

**Keywords:** Trypanosoma cruzi, Chagas disease, GRADE, Screening, Pregnancy, Systematic review

## Abstract

**Background:**

Chagas disease has spread beyond its original borders on the American continent with migration. It can be transmitted from mother to child, through organ transplantation and transfusion of blood and blood products. It is necessary to determine when to screen for this infection.

**Aim:**

Our objective was to evaluate the appropriateness of screening for *Trypanosoma cruzi* infection in Latin American migrants and their descendants.

**Methods:**

We reviewed the literature using rigorous criteria. The quality of evidence was ranked according to the GRADE classification. An evidence to decision framework was adopted to provide information on the most relevant aspects necessary to formulate recommendations.

**Results:**

The 33 studies evaluated revealed a prevalence of *T. cruzi* infection among Latin American migrants in Europe of 6.08% (95% confidence interval (CI): 3.24–9.69; 28 studies). Vertical transmission occurred in three of 100 live births (95% CI: 1–6; 13 studies). The prevalence of cardiovascular disease was 19% (95% CI: 13–27; nine studies), including only 1% severe cardiac events (95% CI: 0–2; 11 studies). The overall quality of evidence was low because of risk of bias in the studies and considerable heterogeneity of the evaluated populations. The recommendations took into account economic studies on the value of screening strategies and studies on acceptability of screening and knowledge of the disease in the affected population.

**Conclusions:**

We identified five situations in which screening for *T. cruzi* infection is indicated. We recommend screening persons from endemic areas and children of mothers from these areas.

## Introduction

Chagas disease is endemic in 21 countries in Latin America and is present from the south of the United States (US) to the north of Argentina and Chile. Transmission in these countries is mainly vectorial, although migration to cities, vertical transmission and blood donations have enabled it to extend from rural to urban areas. Estimations from the year 2010 show that 5,742,167 persons are infected with *Trypanosoma cruzi*. Of these, 62.4% live in the Southern Cone, with an at-risk population of 70.2 million people, 38,593 new cases per year (8,668 congenital cases) and 12,000 related deaths per year. Furthermore, Chagas disease continues to be the leading cause of cardiomyopathy in Latin America and is, according to the World Health Organization (WHO), a ‘forgotten’ or ‘neglected’ disease [1,2].

In Europe, Chagas disease is not monitored systematically, although available data suggest that prevalence rates are high in some countries. The highest numbers of cases in Europe are found, in order of prevalence, in Spain, Italy, the Netherlands, the United Kingdom, Germany and France [3]. According to one study, 4,290 cases of Chagas disease were registered among Latin American immigrants in nine European countries in 2009, with a prevalence of 1.3 cases per 1,000 migrants from endemic countries. In addition to potential underdiagnosis, prevalence among undocumented immigrants could be higher [4].

Spain is home to the largest number of infected persons from Latin America and has the highest number of confirmed cases of Chagas disease in Europe (75% of the total). Prevalence differs for each group of immigrants by country and specific region of origin. Depending on the type of study and population analysed, the highest prevalence rates are found among immigrants from Bolivia (10–40%). Rates are lower in blood donors and pregnant women and higher in patients at reference centres where Chagas disease is screened for [5-9]. Some 53,000 potential carriers were thought to be living in Spain in 2009, and the rate of potentially infected blood donations ranged from 0.02 to 2.35 per 1,000 inhabitants, with wide variations between Autonomous Communities [10]. According to a more recent study and data from the 2011 census and after applying values reported by Basile et al. [4], there are 68,636 *T. cruzi*–infected Latin American immigrants in Spain, of whom 22,100 are women of reproductive age [11].

In endemic areas, *T. cruzi* is vector-borne (reduviid bug). It can also be transmitted vertically, through blood and organ donation and, more rarely, oral contact. Migration has led the disease to spread beyond its original borders, so that it can now be transmitted from mother to child in non-endemic countries or if blood donations and transplants are not monitored [2,12].

Chagas disease has an acute phase, which, if left untreated, progresses to a chronic phase during which patients can develop cardiac, digestive or neurological involvement. This occurs in 30–40% of infected persons 10–30 years after the acute phase and is a major cause of morbidity and mortality. Treatment with antiparasitic agents is effective in the acute phase, in reactivations in immunosuppressed patients, in the chronic phase (especially before the age of 18 years) and in the prevention of vertical transmission [2,12-14]. Although, during the late chronic phase, trypanocidal treatment can be indicated in adult patients with chronic *T. cruzi* infection and no specific organic damage [13], its effectiveness is highly questionable in cases of visceral involvement [2,12-14].

Given that Spain is a country with a high prevalence of *T. cruzi* infection and because early detection of this disease is advantageous (better response to treatment, prevention of materno-fetal transmission and of late organ complications), the Spanish Society of Infectious Diseases and Clinical Microbiology (Sociedad Española de Enfermedades Infecciosas y Microbiología Clínica; SEIMC) decided to draft guidelines on screening for this parasitosis in immigrants and refugees. Here, we present the methodology, results and recommendations of this initiative.

## Methods

We performed a systematic review of the literature to determine whether screening had an impact on a series of relevant outcomes of interest for decision making. We then developed a working framework to formulate explicit, reasoned recommendations.

### Review of the scientific literature

The Panel, which comprised the authors of the study, reviewed the scientific literature according to a protocol following methodological guidelines from the Cochrane Collaboration [15] and reported its findings according to the Preferred Reporting Items for Systematic Reviews and Meta-Analyses (PRISMA) statement [16].

### Clinical question

In order to draw up the recommendations, the Panel asked the following clinical question: Should immigrants and refugees be screened for *T. cruzi* infection?

In order to answer the question, the Panel identified the following outcomes of interest: (i) frequency of vertical transmission, transmission by blood/blood product transfusion and transmission by transplant, (ii) mild to moderate organ involvement (defined as the presence of heart failure or left ventricular ejection fraction < 50%, electrocardiogram abnormalities or dysphagia/constipation), (iii) severe organ involvement (defined as hospitalisation because of heart failure, need for a pacemaker or diagnosis of megasyndrome), (iv) *T. cruzi* infection, (v) indications for a trypanocidal drug, (vi) mortality, (vii) organ involvement of any type after diagnosis and (viii) quality of life.

### Inclusion criteria

Given the lack of clinical trials that compare the impact of screening for *T. cruzi* infection with not screening for it, we considered descriptive studies on screening in which a denominator could be established for the reference population. If this was not possible, we considered case series that described the clinical condition of patients with the disease. The population of interest comprised immigrants or refugees older than 14 years from endemic areas, except in the case of neonatal screening where we also took into consideration minors born to mothers at risk of vertical transmission. We took into account studies that reported experience of screening for *T. cruzi* in non-endemic countries in Europe. The studies had to provide clear detail, namely, test used and setting in which it was performed. By definition, the test is performed in asymptomatic patients.

### Literature search

A search strategy was designed to obtain relevant studies from MEDLINE (accessed via PubMed) and EMBASE (accessed via Ovid) up to October 2018. The strategy combined text words and controlled vocabulary from sources related to screening and *T. cruzi*, with no major limits for year of publication, language or country (Supplement 1). The results of the search were recorded in an EndNoteX2 database to coordinate the eligibility and availability of the studies. The results of the search were complemented with additional references provided by the Panel and based on references from the key studies. The investigators of the original studies were not contacted.

### Data extraction

The Panel retrieved a series of descriptive characteristics from each included study and recorded them on a predefined data extraction form. In addition to the objective and study design, we defined the context, number of participants and their origin, the test used to determine the disease, the criteria for positivity and all relevant specific information from any subgroup of interest (e.g. pregnant women). We also obtained numerical data that made it possible to calculate the prevalence or cumulative rate for each of the outcomes of interest, with their respective confidence intervals (CI) when available.

### Risk of bias

Bias affecting the observational (cross-sectional) studies included was evaluated based on 10 items which assessed the internal and external validity of the studies [17]. The resulting classification defined the overall risk of bias as low, moderate or high. The information obtained from the evaluation of risk of bias was incorporated in the process applied to classify the quality of evidence.

### Data analysis and evidence synthesis

We performed a narrative synthesis of results for each predefined outcome. When prevalence data were available, we calculated pooled estimates with their respective 95% CI using the Freeman-Tukey double arcsine transformation [18]. All tests were two-tailed. We used the random effects model of DerSimonian and Laird to evaluate heterogeneity between studies based on an indicator of variation between studies attributed to heterogeneity rather than to chance (I^2^) [15]. We constructed forest plots showing the point prevalence and the 95% CI of the individual studies, as well as pooled estimates with the 95% CI, both for the total population and for subgroups of interest (e.g. participants’ origin, blood donors and pregnant women). The analyses were performed using the Metaprop command of Stata 12.0 (Stata Statistical Software: Release 12. College Station: StataCorp LP).

The quality of evidence was classified as high, moderate, low or very low in accordance with the methodological guidelines of the Grading of Recommendations, Assessment, Development and Evaluation (GRADE) working group [19]. The process for evaluating the quality of the evidence was included in the summary of findings Tables [20], which also included the main effect estimates for the outcomes of interest.

### Evidence to decision framework

In order to formulate the recommendations in an explicit and reasoned manner, we used an evidence to decision (EtD) framework to inform the Panel of the most relevant aspects necessary for taking decisions and thus making them easy to justify [21].

In addition to the information from the literature review on the outcomes defined in the clinical question, we assessed economic evaluations and studies on perceptions and experiences of the population of interest in order to gather relevant information to inform recommendations on use of resources, values and preferences and acceptability of screening.

## Results

We summarise the main findings from the scientific literature review and the discussion of the Panel for formulating the recommendations. A complete report can be consulted online (Supplement 2).

**Figure 1:**
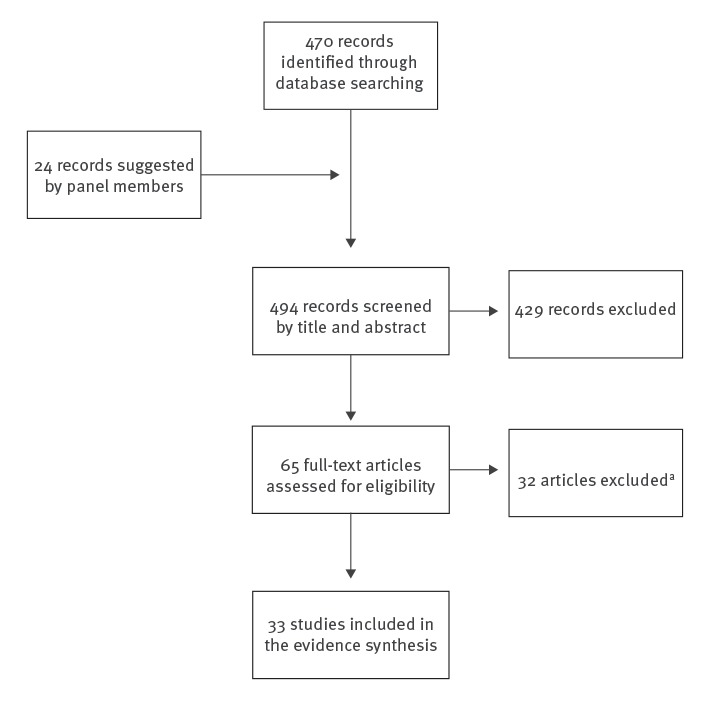
Systematic review about screening of migrants for *Trypanosoma cruzi*, study eligibility, Prisma flow diagram

^a^ Reason for exclusion of full-text articles: 12 narrative review articles; nine conference abstracts; four studies in endemic settings; two cost studies; two duplicates; one case report; one study in a non-eligible setting; one registry report.

The search yielded 470 references. Based on the eligibility criteria, we identified 65 references for which the complete text was reviewed. Of these, we selected 33 studies that fulfilled the inclusion criteria [5-7,9,22-50]. A PRISMA flow diagram (Figure 1) details the eligibility process.

More than half of the studies were prospective (n=20) with a cross-sectional design (n=8) and were carried out in Spain (n=23) and the rest of Europe (n=10). Almost half of the studies focussed on the general migrant population (n=16), while the remainder evaluated vertical transmission (n=13) or included blood donors (n=6). Follow-up varied from a mean of 32 months in prospective studies (range: 12–72 months) to 87 months in retrospective studies (range: 13–148 months). The screening tests varied depending on the study, although they generally involved a combination of an ELISA-based approach and indirect immunofluorescence; the studies also used other, confirmatory tests. The criterion for a positive screening result was generally consistent with the recommendations of the WHO, according to which a case of chronic *T. cruzi* infection is defined by two positive results in different serology tests [12].

### Impact of screening for *Trypanosoma cruzi*

Table 1 summarises the findings of the literature review for the outcomes of interest.

**Table 1:** Summary of outcomes of interest, systematic review about screening of migrants for *Trypanosoma cruzi*

Outcome	Number of studies	Quality of evidence	Impact
Patients with *T. cruzi* infection	26 observational studies [5-7,9,22-43]	Low	1. Total pooled prevalence: 6% (95% CI: 3–10); 2. Among immigrants in general: 13% (95% CI: 7–21; I^2^ = 98.15%) 3. Pregnant women: 4% (95% CI: 2–7; I^2^ = 96.28%) 4. Blood donors: 0.42% (95% CI: 0.03–1.08%; I^2^ = 82.06)
Vertical transmission of *T. cruzi* infection	13 observational studies [6,35-43,46,49,50]	Low	1. Pooled transmission rate: 3 per 100 live births (95% CI: 1–6; I^2^ = 21.40%) 2. Symptoms of infection in 20% (95% CI: 0–53; I^2^ = 32.4%)
Mild to moderate organ involvement: cardiovascular symptoms	9 observational studies [6,7,9,24,29,45-48]	Low	Cardiovascular disease: 19% (95% CI: 13–27%; I^2^ = 88.36%)
Mild to moderate organ involvement: digestive symptoms	9 observational studies [7,24,25,28,29,45-48]	Low	Gastrointestinal abnormalities: 5% (95% CI: 2–11; I^2^ = 89.62%)
Severe organ involvement	11 observational studies [6,7,9,24,25,28,29,45-48]	Low	1. Severe cardiac events: 1% (95% CI: 0–2; I^2^ = 57.9%) 2. Severe gastrointestinal involvement (megasyndrome): 0% (95% CI: 0–1; I^2^ = 41.26%)
Indication for treatment	11 observational studies [6,29,30,35,37,39,43,45-48]	Very low	1. Initiation of trypanocidal therapy: 81% (95% CI: 67–93; I^2^ = 93.25%) 2. End of treatment: 78% (95% CI: 65–89; I^2^ = 88.5%) 3. Treatment-related adverse effects: 47% (95% CI: 32–63; I^2^ = 90.45%)
Mortality	2 observational studies [6,7]	Low	Two reports of sudden cardiac death secondary to Chagas disease

CI: confidence interval.

### Patients with *Trypanosoma cruzi* infection

**Figure 2:**
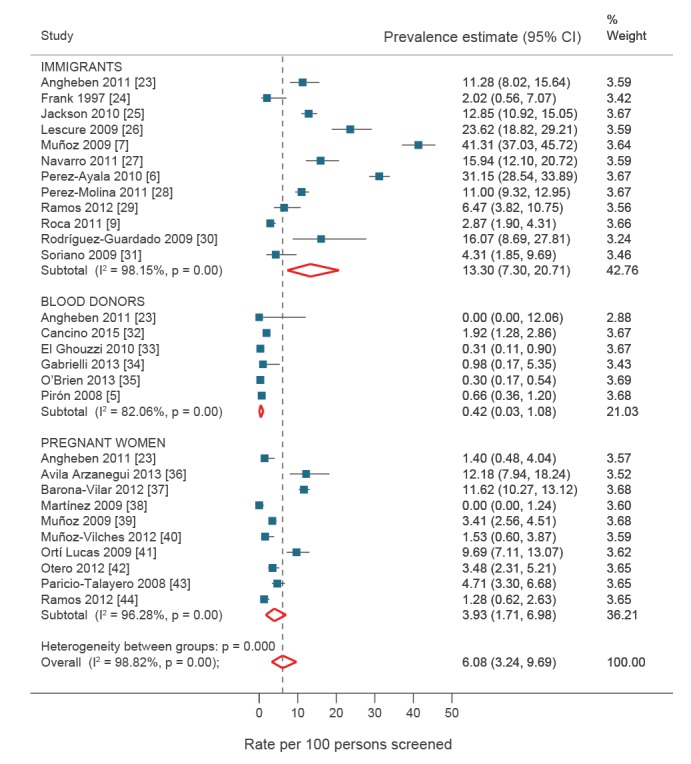
Prevalence of *Trypanosoma cruzi* infection: pooled analysis from studies included in the review

We collected data from 28 observational studies covering 1,441 cases in a population of 19,735 immigrants from endemic areas. The total pooled prevalence of *T. cruzi* infection was 6.08% (95% CI: 3.24–9.69%; I^2^ = 98.82%). Of the total number of cases where it was possible to identify the country of origin (n = 986; from 17 countries), 90% (n = 884) were from Bolivia, followed by Argentina (n = 32; 3.3%), Paraguay (n = 25; 2.5%) and Ecuador (n = 12; 1.22%). Prevalence was systematically higher in Bolivian immigrants than in all other groups. Considerable differences were found in population subgroups according to the setting where screening was performed (Table 1, Figure 2).

CI: confidence interval.

### Vertical transmission of *Trypanosoma cruzi* infection

Vertical transmission was recorded in 27 cases from 13 observational studies covering a total of 502 children born to mothers with chronic *T. cruzi* infection [6,35-43,46,49,50]. The pooled transmission rate was three per 100 live births (95% CI: 1–6).

### Organ involvement

We recorded 343 cases of mild to moderate cardiovascular disease in 1,946 seropositive patients (nine studies), a cumulative prevalence of 19% (95% CI: 13–27%) [6,7,9,24,29,45-48]. Furthermore, nine studies yielded 180 cases with a mild to moderate digestive tract disorder from 1,862 patients (pooled prevalence: 5%; 95% CI: 2–11%) [7,24,25,28,29,45-48].

Nine observational studies yielded a cumulative prevalence of severe cardiac events of 1% (95% CI: 0–2%; 39 events in 1,946 patients) [6,7,9,24,29,45-48]. The estimated cumulative prevalence of megasyndrome was lower (0%; 95% CI: 0–1%; 45 events in 1,935 patients) [6,7,9,24,25,28,29,45-48].

### Indication for treatment

Data from 11 observational studies showed that in 81% of detected cases, treatment was started with a trypanocidal drug (95% CI 67–93%) [6,29,30,35,37,39,43,45-48]. These studies revealed treatment-related adverse effects in 47% of cases (95% CI: 32–63%).

The usefulness of trypanocidal treatment in adults with Chagas disease is questionable. A 2009 meta-analysis showed limited benefit of treatment with benznidazole in chronic disease, with a marginal effect compared with placebo [51]. The only randomised clinical trial comparing benznidazole with placebo revealed no benefit of benznidazole in clinical outcomes (heart disease or death); it reported that *T. cruzi* remained undetectable in PCR in more patients treated with benznidazole than in those who received placebo, although no association with clinical outcomes was observed [52]. Since the study patients had moderate to severe heart disease, we do not know whether benznidazole controls disease in patients with mild or no organ involvement. Furthermore, recent clinical trials on the use of imidazoles (posaconazole or ravuconazole) alone [53,54] or in combination with benznidazole [55] showed these to be less efficacious than benznidazole based on microbiological response markers (PCR for *T. cruzi*). A systematic review on safety of treatment showed that benznidazole was poorly tolerated, with a frequency of adverse effects of 44% and discontinuation of treatment in 11% of patients [56].

Nevertheless, promising alternative regimens based on different doses and durations of treatment with benznidazole are being developed. The objective of trials investigating these regimens is to improve tolerability while maintaining treatment efficacy (Multibenz NCT03191162; BENDITA NCT03378661; BETTY NCT03672487). Preliminary results have demonstrated reductions in the frequency of adverse events, with equivalent percentages of undetectable *T. cruzi* in PCR at 12 months [57].

### Mortality

Two studies reported one case each of sudden death from cardiac causes secondary to Chagas disease [6,7].

### Quality of evidence

The Panel rated the overall quality of evidence for the evaluated outcomes as low. Given the absence of clinical trials that evaluated the efficacy of screening in the population of interest, the included studies were observational (generally cross-sectional), with a prospective or retrospective design and, therefore, a risk of selection bias that could affect confidence in the reported results. The study populations were heterogeneous, resulting in marked statistical heterogeneity for the outcomes of interest in most analyses. However, this variability diminished when studies with well-defined populations (e.g. pregnant women) were evaluated together as a subgroup (Figure 2).

### Recommendations

Box 1 shows an adaptation of the EtD framework based on which the Panel discussed and formulated the recommendations. The complete EtD framework can be consulted in Supplement 2. The Panel subsequently formulated the recommendations included in Box 2.

Box 1 **Evidence-to-decision framework for the formulation of recommendations for screening of migrants for *Trypanosoma cruzi***

**Table:** 

**Clinical question** Should immigrants and refugees undergo screening for *T. cruzi* infection? **Population of interest** Migrants and refugees **Intervention** Screening for *T. cruz*i infection **Comparison** Not screening **Outcomes of interest** Cases of vertical transmission resulting from transfusion or transplant Mild to moderate organ involvement (heart failure or LVEF < 50%, ECG abnormalities or dysphagia/constipation) Severe organ involvement (hospitalisation due to heart failure, need for a pacemaker or diagnosis of megasyndrome) Infection Indication for trypanocidal treatment Mortality Organ involvement of any type after diagnosis Quality of life
**Is the problem a priority?** *Yes* **Research evidence and remarks from the Panel** High prevalence of immigrants from Latin America, especially Bolivia. Between 50,000 and 70,000 persons in Spain are thought to have the disease. Autochthonous transmission occurs in Spain, mainly by vertical transmission. Early detection in children of infected mothers indicates a very high rate of cure. Early detection improves response to treatment. Antiparasitic treatment is more effective in children and adolescents than in adults. It also makes it possible to identify women of reproductive age who have not yet had children (or who have had children and may wish to have more) and in whom treatment blocks vertical transmission. There is the possibility of reactivation in immunosuppressed patients, with severe consequences. Approximately 30% of persons with Chagas disease develop heart disease, with subsequent morbidity and mortality and added cost for the health system. Chagas disease carries a risk of stigmatisation. Better knowledge and normalisation of the disease improves the negative perception that often keeps patients from being diagnosed and treated early.
**How substantial are the desirable anticipated effects?** *Large* **How substantial are the undesirable anticipated effects?** *Moderate* **Research evidence and remarks from the Panel** See Table 1 for a summary of findings for the outcomes of interest. The prevalence of the disease in the study population, the rate of vertical transmission, and the rate of complications—mainly cardiovascular complications—justify screening. In the case of indication for treatment, treatment of chronic infection in children younger than 19 years is very useful, while in adults, it should be questioned owing to the lack of quality studies. The efficacy of treatment in chronic infection may be questionable, especially for patients with moderate to severe cardiopathy. The marginal effect of treatment for adults in this phase of the disease could be due to considerable differences between studies [51-55,68]. While the tolerability of parasiticidal treatment is good in children, it is important to highlight the poor tolerability of treatment in adults: up to 44% of patients treated with benznidazole experience adverse effects, which have an impact on discontinuation of treatment (11% of all treated patients) [56]. The adverse effects of screening are minimal, i.e. those associated with blood sampling and the potential psychological consequences of having the disease, which may lead to stigmatisation. However, treatment with trypanocidal agents led to a high rate of adverse effects (on occasion up to 50%), some of which were severe. There is little evidence on the real cure rates in adult patients with chronic disease, which may be around 30% [68].
**What is the overall certainty of the evidence of the effects?** *Low* **Research evidence and remarks from the Panel** The studies evaluated for this question are observational (generally cross-sectional) with a prospective or retrospective design. The populations evaluated are heterogeneous. The quality of the results of these studies can be affected by selection bias. Furthermore, most of the combined analyses for the outcomes of interest revealed a very notable rate of statistical heterogeneity. However, this variability decreased when studies with well-defined populations (e.g. pregnant women) were evaluated together or when variability was attributed to estimations from few studies. Therefore, in most studies it was not considered a reason for reduced quality of evidence.
**Is there major uncertainty about or variability in how much people value the main outcomes?** *There may be major uncertainty or variability.* **Research evidence and remarks from the Panel** A study on Bolivian women with Chagas disease in Madrid revealed relevant aspects of their knowledge of and attitudes towards the disease. Most knew that the disease was transmitted by a vector, could be asymptomatic, and could lead to severe complications such as sudden death and heart failure. While there is some confusion over treatment, the women know that it is available [69]. The study included women from a country where Chagas disease was highly prevalent. There may be major differences between people from different countries.
**Does the balance between desired and undesired effects favour the intervention or the comparison**? *The balance between desired and undesired effects favours the intervention.* **Research evidence and remarks from the Panel** The expected benefit is centred on three basic aspects: Intervention prevents vertical transmission, with treatment offered after serology-based diagnosis in women of reproductive age from endemic areas. Furthermore, detection of the disease in a pregnant woman enables early diagnosis and treatment of the newborn, thus ensuring cure rates close to 100% during the first year, with very few adverse effects. Early diagnosis and treatment of the disease could prevent mainly cardiovascular complications, although evidence is scarce. The risks associated with treatment can be controlled to a large extent with appropriate follow-up at specialised clinics during the administration period. Screening of donors to prevent transmission through blood products and organs is addressed in current Spanish legislation (Royal Decree 1088/2005, dated 16 September 2005 (BOE-A-2005–15514))
**Is the intervention acceptable to key stakeholders (population, professionals)?** *Probably* **Research evidence and remarks from the Panel** The participants in the study on Bolivian women living in Madrid showed a certain degree of indifference and a lack of understanding of the risk of contracting the disease [69]. Participants who presented symptoms or had relatives with symptomatic disease expressed their concern for the more severe complications of the disease and the possibility of vertical transmission. In addition, fear of rejection by their social circle because of the disease was detected. In a similar study in the US, participants also expressed their concern about the lack of knowledge of the disease by health professionals and about more logistic aspects, such as difficulties reaching health centres or having sufficient time to attend an appointment. Of note, the participants in this study were at risk of social exclusion [70]. Stigmatisation because of the disease in specific groups should be taken into consideration when screening. Education, knowledge of the disease and easy access to diagnosis and treatment are necessary in order to overcome this problem.
**Does the cost-effectiveness of the intervention favour the intervention or the comparison?** *They probably favour the intervention.* **Research evidence and remarks from the Panel** An economic evaluation [11] evaluated the most efficient strategy for controlling Chagas disease among Latin American immigrants living in Spain. The study showed that not screening was more expensive and less effective and was dominated by other strategies from the societal perspective and from the perspective of the Spanish National Health System (SNHS). One of the most efficient strategies from both perspectives was screening of pregnant women, their newborns and first- and second-degree relatives of disease-positive mothers. From the SNHS perspective, moving from the strategy of screening ‘mother and newborn’ to the strategy of including ‘relatives of a disease-positive mother’ would involve a mean increase of EUR 301 per patient and QALY gained. In addition, moving from a strategy of ‘relatives of a disease-positive mother’ to ‘relatives of a disease-negative mother’ would involve a mean increase of EUR: 30,844 per patient and QALY gained. The parameters whose modification had the greatest effect on the results were efficacy of treatment of chronic disease and disease prevalence. We observed that it could prove efficient to extend a programme of this type to relatives of disease-negative mothers if the efficacy of treatment increased or the programme targeted the population at greatest risk. Results were recently published for an economic evaluation of systematic screening for Chagas disease in the Latin American population receiving primary care in Europe [71]. The evaluation consisted of a decision-making approach based on a Markov model in which the impact of systematic screening (all asymptomatic Latin Americans seen at primary care setting, treatment and follow-up of positive cases) was compared with screening and treatment of symptomatic persons. The model was based on a simulation in a cohort of 100,000 Latin American citizens, with a *T. cruzi* infection rate of 4.2% (95% CI: 2.2–6.8) and a maximum of 5 years of treatment in positive cases. The results of the analysis according to the probabilistic models revealed a total cost of systematic screening of EUR 32,163,649 (95% CI: 31,263,705–33,063,593) compared with EUR 6,904,764 (95%: CI: 6,703,258–7,106,270) for screening of symptomatic persons. The difference in QALY gained was 4,758.62 (95% CI: 4,618.42–4,898.82) for systematic screening compared with testing only symptomatic patients, with an incremental cost (ICER) of EUR 6,840.75 (95% CI: 6,255.75–7,425.75) for each QALY gained, treatment efficacy of 20% and EUR 4,243 for each QALY gained when efficacy was estimated to be 50%. Therefore, systematic screening in primary care would prove cost-effective, even in scenarios with a very low disease prevalence (0.05%). Another economic evaluation compared a decision-making model for screening the newborn or the mother against the alternative of no screening [72]. All of the models revealed screening to be the dominant (clinically superior and cost saving) situation compared with not screening. The ICER for the screening in the newborn was EUR 22 per QALY gained compared with EUR 125 per QALY gained in the case of not screening. In the case of mothers, the ICER was EUR 96 per QALY gained compared with EUR 1,675 per QALY gained for not screening. Screening strategies were cost-effective, even in settings with a higher prevalence of Chagas disease (0.9%) and lower risk of vertical transmission (2.2%), where the ICER increased by only EUR 37.50 per QALY gained for both strategies. It would be necessary to perform studies on the costs for the health system of a patient with symptomatic Chagas disease, especially when this involved heart disease.

CI: confidence interval; ECG: electrocardiogram; ICER: incremental cost-effectiveness ratio; LVEF: left ventricular ejection fraction; QALY: quality-adjusted life year.

Box 2 **Recommendations on the screening of migrants for *Trypanosoma cruzi***

**Table:** 

**Should immigrants and refugees be screened for *T. cruzi* infection?** Screening for Chagas disease is recommended in pregnant women (from endemic areas or daughters of women from these areas). Strong recommendation. Screening for Chagas disease is recommended in women of reproductive age from endemic areas who wish to become pregnant and daughters of women from these areas. Strong recommendation. Screening for Chagas disease is recommended in blood and organ donors (from endemic areas or children of mothers from these areas). Strong recommendation. Screening for Chagas disease is suggested in immunosuppressed patients or those at risk of immunosuppression (persons from endemic areas or children of mothers from these areas), ideally before immunosuppression or at diagnosis of HIV infection. Conditional recommendation. It has been suggested that asymptomatic adults from endemic areas should receive help in making a joint decision on the possibility of screening for Chagas disease. They should be informed about the characteristics and consequences of the disease, the advantages and disadvantages of treatment and the limited benefit of treatment in the case of a latent infection. Conditional recommendation.

HIV: human immunodeficiency virus.

### Justification

Recommendations have been formulated in favour of screening owing to the high prevalence of *T. cruzi* infection among immigrants from Latin America, especially Bolivia. Furthermore, the possibility of autochthonous transmission, mainly vertical transmission, is notable in Spain. Given that the response to trypanocidal treatment is greater than 90% during the first year of life, early detection in the children of affected mothers provides huge benefits. A strong recommendation has been formulated for women of reproductive age who wish to become pregnant, since this situation is considered to be an opportunity for preventing transmission to future children, taking into account the rate of maternofetal transmission of Chagas disease (3–7%) [2,13]. Transmission is more frequent when PCR in blood is positive than when it is negative.

The Panel also considered that blood or organ donation is a situation where identification of people with infection could be particularly relevant for preventing transmission and providing appropriate treatment for positive cases.

Immunosuppressed patients are at risk of reactivation of disease and progressing to a more severe clinical condition; therefore, the potential benefit of treatment is greater in this subgroup of patients. Ideally, screening should be performed before immunosuppression is detected, although it is not always possible to know this in advance. Therefore, screening can also be performed once immunosuppression has been confirmed. However, in the case of patients from non-endemic countries, it is not recommended to repeat serology when the result is negative. The following patient groups are considered as immunosuppressed: patients taking immunosuppressants (e.g., cytostatic agents, corticosteroids) at doses > 1 mg/kg for more than 1 month, biological drugs with potent immunosuppressive action or other types of immunosuppressive antineoplastic chemotherapy, transplant recipients, HIV-infected patients and patients with haematological cancer.

In the case of asymptomatic adults, a conditional recommendation was formulated on shared decision making with the patient, given the beneficial effect of treatment and the low quality of evidence in this patient group. However, the Panel considered the option of screening involving the patient in diagnosis and treatment for several reasons: (i) poor visibility and knowledge of the disease, (ii) the major psychological impact of the disease on the potentially affected population and (iii) the fact that the benefit of treatment in this group of patients would be strictly on an individual basis. In non-endemic countries, patients are screened only once since there is no risk unless they return to an endemic country for long periods. Therefore, a pregnant woman with a negative result for *T. cruzi* in serology testing does not require further screening in subsequent pregnancies if she remains in Spain.

## Discussion

The Panel of the SEIMC reviewed the literature to determine the appropriateness of screening for *T. cruzi* infection in immigrants and refugees and used explicit criteria to formulate a series of reasoned recommendations for this purpose. Following this rigorous and systematic methodology, we identified five situations where screening for *T. cruzi* infection is indicated. The recommendations are justified by the high prevalence of this problem in immigrants from Latin America and a notable rate of autochthonous transmission in Spain, mainly owing to vertical transmission. 

*Trypanosoma cruzi* infection is the most common imported parasitic disease in Spain. Nevertheless, there remains much room for improvement with respect to diagnosis. A Royal Decree from 2005 set out the minimum conditions and technical requirements for blood donation (RD 1088/2005). This law covered screening for *T. cruzi* infection. Similarly, screening for the infection has been part of transplant programmes for several years. However, it is not yet part of routine practice in protocols for pregnant women in most Autonomous Communities, despite the fact that recommendations are in place [58,59]. Likewise, most countries in the European Union do not have legislation regulating screening for Chagas disease in pregnant women from endemic areas [60]. Furthermore, the prevalence of *T. cruzi* infection is clearly higher in populations that may also be infected by HIV, hepatitis B virus and syphilis and that do in fact undergo regular screening [61,62]. This disease burden has been observed in studies on the seroprevalence of *T. cruzi* and on the serological profile of pregnant women, with a prevalence of 1.75–5% [42,63,64]. The results of our review show a pooled vertical transmission rate of three per 100 live births, which is lower than reported in another systematic review (4.7 per 100 live births) [65]. Similarly, screening should be included in health programmes aimed at immigrant children or children of immigrants from endemic areas [11,42].

The Panel used a systematic and explicit methodology to formulate the recommendations. While we were unable to identify a clinical trial that could provide data on the relevance of screening for *T. cruzi* infection, we evaluated a large number of studies from which we were able to retrieve data on the outcomes of interest based on the experience of other authors. Using data from the literature review, the Panel created an EtD framework that enabled an explicit and reasoned assessment of the main criteria for formulating recommendations [21]. When it comes to implementing the recommendations, this process makes it possible for users to evaluate all the aspects and opinions that the Panel took into consideration during the formulation process.

The paucity of relevant literature and its limited quality with respect to the decision-making process in some cases (e.g. asymptomatic adults) could limit the proposal for recommendations in these specific situations or open a debate on their relevance. In any case, the process for using the available information and justifying the different opinions of the Panel validates the recommendations, since these explicitly reflect all the considerations taken into account, even those that were more subjective.

Another potential limitation of this study is that we did not include studies conducted explicitly in children younger than 14 years. Most of the studies in non-endemic countries are performed in migrants and refugees older than 14 years (younger people are rarely included because of difficulties with informed consent). Nevertheless, and given that Chagas disease can be transmitted vertically, studies including screening of neonates and of minors born to mothers at risk of vertical transmission, were also taken into consideration.

While Latin American immigrants in Spain account for 24.9% of all foreigners (Bolivians for 1.9%) and the greatest increases in non-EU foreign nationals in 2018 were recorded among citizens of endemic countries such as Venezuela and Colombia [66], there are no protocols for screening for *T. cruzi* infection in most Spanish Autonomous Communities. Despite agreement in Europe on the need for screening to control transmission in blood banks and in the transplantation setting, there is considerable uncertainty with regard to congenital transmission and the provision of care to Latin American immigrants. In fact, this uncertainty is so widespread that other similar guidelines (e.g. guidelines from the European Centre for Disease Prevention and Control on screening and vaccination for infectious diseases in newly arrived migrants within the European Union and European Economic Area) have not addressed this issue [67].

The recommendations made by the SEIMC can serve as a guide for the development of protocols and help health professionals make decisions on screening for this disease in clinical practice in other countries.

## Conclusions

The use of a rigorous methodology enabled us to formulate recommendations in an explicit and reasoned way by ensuring that during the formulation process, the Panel had information on the most important criteria for decision making.

As for the usefulness of screening immigrants and refugees for *T. cruzi*, the Panel of the SEIMC identified a series of situations in which screening would be indicated. The recommendation is aimed at people who could benefit most from the diagnosis (children and adolescents up to 19 years of age, young women of reproductive age and pregnant women), mainly owing to the high prevalence of this infection in immigrants (especially Bolivians) and the possibility of preventing autochthonous transmission.

## Supplementary Data

32000 Supplement1

## Supplementary Data

961000 Supplement2
