# Supplementary material for: Screening for Trypanosoma cruzi infection in immigrants and refugees: Systematic review and recommendations from the Spanish Society of Infectious Diseases and Clinical Microbiology
Source: Euro Surveill. 2020 Feb 27;25(8):1900393. doi: 10.2807/1560-7917.ES.2020.25.8.1900393 (PMC7055039; doi:10.2807/1560-7917.ES.2020.25.8.1900393)
Supplement: Supplement1 [file 19-00393_Supplement1_PEREZ_MOLINA.pdf]

ONLINE SUPPLEMENT I

SEARCH STRATEGY DESIGNED TO OBTAIN RELEVANT STUDIES FOR THE REVIEW

MEDLINE (PubMed) Oct 2018

#1	"Emigration and Immigration"[Mesh]	24604
#2	"Emigrants and Immigrants"[Mesh]	10947
#3	"Transients and Migrants"[Mesh]	10650
#4	"Refugees"[Mesh]	9203
#5	immigr*[tiab]	30582
#6	emigr*[tiab]	7422
#7	migrat*[tiab]	283296
#8	migrant*[tiab]	17294
#9	diaspor*[tiab]	704
#10	refugee*[tiab]	9102
#11	asylum seek*[tiab]	1419
#12	#1 OR #2 OR #3 OR #4 OR #5 OR #6 OR #7 OR #8 OR #9 OR #10 OR #11	341580
#13	"Early Diagnosis"[Mesh]	43425
#14	"Mass Screening"[Mesh]	120866
#15	screen*[tiab]	664860
#16	tested[tiab]	874658
#17	testing[tiab]	488277
#18	prevention and control[tiab]	18782
#19	preventi*[ti]	201160
#20	earl[ti]	169
#21	early[ti]	267294
#22	#13 OR #14 OR #15 OR #16 OR #17 OR #18 OR #19 OR #20 OR #21	2340870
#23	"Trypanosoma cruzi"[Mesh]	11174
#24	Trypanosoma cruzi[tiab]	13099
#25	T. cruzi[tiab]	7962
#26	#23 OR #24 OR #25	14811
#27	#12 AND #22 AND #26	169

OVID Embase 1974 to 2018 October

1 exp migration/ (42837)
2 exp migrant/ (33552)
3 exp refugee/ (11890)
4 (immigr* or emigr* or migrat* or migrant* or diaspor* or refugee* or asylum seek*).ti,ab. (417669)
5 1 or 2 or 3 or 4 (436401)
6 exp early diagnosis/ (100134)
7 exp mass screening/ (225168)
8 (screen* or tested or testing).ti,ab. (2548829)
9 (preventi* or earl or early).ti. (588436)
10 6 or 7 or 8 or 9 (3195672)
11 exp Trypanosoma cruzi/ (14808)
12 (Trypanosoma cruzi or T cruzi).ti,ab. (15236)
13 11 or 12 (17656)
14 5 and 10 and 13 (301)

"This supplementary material is hosted by Eurosurveillance as supporting information alongside the article "Screening for *Trypanosoma cruzi* infection in immigrants and refugees: Systematic review and recommendations from the Spanish Society of Infectious Diseases and Clinical Microbiology (SEIMC)", on behalf of the authors, who remain responsible for the accuracy and appropriateness of the content. The same standards for ethics, copyright, attributions and

permissions as for the article apply. Supplements are not edited by Eurosurveillance and the journal is not responsible for the maintenance of any links or email addresses provided therein."
