# Supplementary material for: Screening for Trypanosoma cruzi infection in immigrants and refugees: Systematic review and recommendations from the Spanish Society of Infectious Diseases and Clinical Microbiology
Source: Euro Surveill. 2020 Feb 27;25(8):1900393. doi: 10.2807/1560-7917.ES.2020.25.8.1900393 (PMC7055039; doi:10.2807/1560-7917.ES.2020.25.8.1900393)
Supplement: Supplement2 [file 19-00393_Supplement2_PEREZ_MOLINA.pdf]

ONLINE APPENDIX II – TECHNICAL REPORT COMPLETED BY THE PANEL

Spanish Society of Infectious Diseases and Clinical Microbiology

Clinical Practice Guidelines on Screening for Infectious Diseases in Immigrants and Refugees

Clinical Question: Should immigrants and refugees be screened for *Trypanosoma cruzi* infection?

"This supplementary material is hosted by Eurosurveillance as supporting information alongside the article "Screening for Trypanosoma cruzi infection in immigrants and refugees: Systematic review and recommendations from the Spanish Society of Infectious Diseases and Clinical Microbiology (SEIMC)", on behalf of the authors, who remain responsible for the accuracy and appropriateness of the content. The same standards for ethics, copyright, attributions and permissions as for the article apply. Supplements are not edited by Eurosurveillance and the journal is not responsible for the maintenance of any links or email addresses provided therein."

SCIENTIFIC EVIDENCE REPORT

1. METHODS

1.1. Review of the scientific literature

The literature review designed to inform the clinical question was consistent with the methodological guidance applied in systematic reviews. The methodological guidelines used were those of the Cochrane Collaboration (Higgins 2011), and the PRISMA statement was followed to report its findings (Moher 2009). A rapid review of the literature was performed, since this made it possible to perform methodologically timely and rigorous reviews, while simplifying some of the stages of systematic reviews (e.g., prioritizing the inclusion of other published systematic reviews) (Tricco 2015). The most relevant methodological aspects for drafting a literature review are outlined below.

1.2. Clinical question

Should immigrants and refugees be screened for *Trypanosoma cruzi* infection?

Table 1. Structured clinical question

**Population**	Asymptomatic adult immigrants (>14 years) from endemic areas
**Procedure**	Screening for *T. cruzi* infection
**Alternative**	Not screening for *T. cruzi* infection
**Outcomes of interest***	• Patients with infection<br>• Cases of vertical transmission through transfusion or transplant<br>• Mild-to-moderate organ involvement (presence of heart failure or LVEF <50%, ECG abnormalities, or dysphagia/constipation)<br>• Severe organ involvement (hospitalization for heart failure, need for a pacemaker, or diagnosis of megasyndrome).<br>• Organ involvement of any type after diagnosis.<br>• Indication for trypanocidal treatment, adherence to treatment, treatment-related adverse effects.<br>• Quality of life<br>• Mortality

*The outcomes of interest were identified by the Panel.

1.3. Eligibility criteria

First, we considered other literature reviews to be the main source of studies with the data necessary for an evidence synthesis.
The inclusion criteria taken into consideration for this clinical question were as follows:
Study area: Countries that were not endemic for *T. cruzi*, preferably in Europe.
Intervention: The intervention had to be clearly detailed with respect to the test used and the setting in which the test was performed. By definition it is performed in asymptomatic patients.
Design: Descriptive studies where it is possible to determine a denominator or reference population, or if this is not possible, a series of cases in which the patients present the symptoms of this disease.
Population: Immigrants from endemic areas aged >14 years. In the case of neonatal screening, the population also includes persons born to mothers at risk of vertical transmission. Languages: English, Spanish, French.
We did not set limitations with respect to the date of publication.

The exclusion criteria were as follows:
Study area: Endemic countries, because the risk of transmission and prevalence do not reflect the reality of countries where there is no natural vector.

Type of publication: Review articles, case reports, posters, congress abstracts, and non–peer-reviewed publications.
Unit of study: Other than patients, blood products.
Screening method: Method used and/or positivity criteria not specified.

1.4. Search strategy

In order to obtain studies that are relevant to the clinical question, we designed a search in MEDLINE (accessed via PubMed) and EMBASE (accessed via Ovid). The search strategy included 3 microorganisms (*Schistosoma spp., Trypanosoma cruzi*, and *Strongyloides* spp.) and was implemented in October 2018. The complete search algorithm is presented in Appendix I.
The results of the search were managed from an EndNoteX2 database to coordinate the eligibility and availability of the studies.
The search results were complemented with additional references contributed by the Panel and based on the references from the main studies.

1.5. Evidence synthesis

Risk of bias: The risk of bias of the clinical trials was evaluated as proposed by the Cochrane Collaboration (Higgins 2011). We specifically evaluated whether the studies were subject to selection bias (by assessment of the generation of the randomization sequence and masking), detection bias (by assessment of blinding of the evaluation of the outcomes of interest), and bias due to loss to follow-up (by assessment of the accuracy with which the losses to follow-up were described and the similarity between these in the study arms). If any of these types of bias was present, we considered the trial as being at high risk of bias. We also evaluated performance bias (by assessment of blinding of the investigators and participants) and bias in reporting of outcomes of interest (by assessment of potential discrepancies between the outcomes stated in the trial protocols and the publication of results in scientific journals).
The tool used in observational prevalence studies was that of Hoy et al (Hoy 2012), who took into account 10 items, based on which they evaluated the internal and external validity of the study and categorized it as having a low, moderate, or high risk of bias. The information obtained from the evaluation of the risk of bias was integrated in the process of classification of quality of evidence.

Synthesis of outcomes: We performed a qualitative and quantitative synthesis of the results reported based on the studies obtained in the literature search. We present a summary of the main results before providing a detailed description.
In the case of original studies, we retrieved the main data that enabled us to describe the characteristics of the studies and to calculate the effect estimator.
In all cases, we reported the result of the literature search and the process for determining the eligibility of the studies considered for the review.
All questions contained a short mention of the current status of disease burden in the target population of the clinical question, both in Europe and in Spain, based on official data or key studies identified in the search.
We provide the main characteristics of the studies included and describe the main findings of the review for each of the outcomes of interest, together with a discussion on the evaluation of the quality of the evidence.
We used a data collection form covering the following variables of interest:

- Country, year of publication, and setting for screening, i.e., place and circumstances of screening.
- Objectives and scope, described in the study as the primary objective.
- Study period: start and end dates, retrieval of clinical histories, and case collection.
- Characteristics of the study population: total number of participants, sex, age, and migratory status at screening.
- Origin: with emphasis on the most frequent geographic areas and/or countries of origin.
- Screening test: with detail on the technique(s) used and the cut-off points for considering a result to be positive.
- Results of screening, main findings according to the outcomes prioritized by the Panel.

Data analysis: A narrative synthesis of the results was made for each outcome. When the prevalence data became available, the grouped prevalence estimates were calculated with their 95% confidence intervals (CI)

using the Freeman-Tukey double arcsine transformation (Nyaga 2014). The Der Simonian and Laird random effects model was used. Heterogeneity was evaluated using I^2, which indicates variation between studies attributed to heterogeneity rather than to chance (Higgins 2003). A stratified analysis was performed by subgroups. The data collected in this review were the estimations of prevalence with no interventions; therefore, the publication bias was expected to be insignificant. The forest plot shows the point prevalence and the 95%CI of the individual studies, as well as the grouped estimations and the 95%CI for all the strata. All tests were 2-tailed. The analyses were performed using the *metaprop* command in Stata 12.0 (StataCorp, 2011. *Stata Statistical Software: Release 12.* College Station, TX: StataCorp LP).

The prevalence by country of origin was calculated by including the results corresponding to cases detected in persons from endemic countries based on studies that reported these data according to the country of origin.

A case of chronic *T. cruzi* infection was defined according to the recommendations of the World Health Organization (WHO), which indicate that this condition is confirmed using 2 serology tests based on different principles.

Symptoms (morbidity) were analyzed by including in the meta-analysis only those studies of immigrants in general, since findings from these studies are considered to have greater external validity. In other populations, these data are set out in narrative form or are presented in the attached tables. The denominator used in the studies that reported the symptoms is the total number of patients who underwent the evaluation, in terms of cardiovascular and/or digestive symptoms. In any other case, the denominator is the total number of cases.

Quality of evidence: We classified the quality of evidence as high, moderate, low, or very low according to the methodological guidelines of the GRADE system (Guyatt 2008, Guyatt 2011). The process for evaluating the quality of evidence is shown in detail in the Summary of Findings, which also includes the main effect estimates for the outcomes of interest.

Evidence on the use of resources and patient values and preferences: Based on the search strategy described above, an attempt was made to identify economic evaluations that were relevant for the question, as well as studies on variability in the importance given by patients to the outcomes of interest.

1.6. Justification of the evidence for the recommendation

In order to present those aspects that contributed to the formulation of the recommendation and the grading of its strength, we developed a framework that made it possible to provide explicit judgments on the process that informed the Panel during the formulation of recommendations. This framework was based on the guidelines developed in the GRADE Working Group "evidence-to-decision framework” (EtD; www.guidelinedevelopment.org/handbook).

2. MAIN REVIEW FINDINGS

Search results

The search strategy yielded 1,228 references. A full-text review was performed for 72 references, from which 33 studies were selected. These reported 35 experiences of screening for *T. cruzi* in immigrants from endemic areas.

Most studies were performed in Spain; 16 (46%) were performed in immigrants who consulted at an outpatient clinic. The studies were prospective and descriptive. Depending on the setting, 6 studies were identified in blood donors and 13 in pregnant women undergoing antenatal screening.

Disease burden: Chagas disease in Europe and Spain

Chagas disease is endemic in 21 Latin American countries, where is mainly transmitted by vectors and from mother to child. In Europe, Chagas disease is not systematically monitored, although available data suggest that prevalence rates are quite high in some countries. Estimates show that for the year 2011 in Spain, there were 68,636 *T. cruzi*–infected persons from Latin America, of whom 22,100 were women of reproductive age (Imaz 2015).

Review findings for the outcomes of interest

Patients with infection: Prevalence

The total pooled prevalence was 6% (95% CI, 3%-10%; I^2, 98.8%) (1,441 cases in 19,735 immigrants, 28 studies). In 90% of cases the patient was from Bolivia. Prevalence was 13% (95% CI, 7%-21%; I^2, 98.15%) among immigrants in general, 4% (95% CI, 2%-7%; I^2, 96.28%) in pregnant women, and 0.42% (95% CI, 0.03%-1.08%; I^2, 82.06) in blood donors.

Prevalence by country of origin was as follows: Bolivia, overall prevalence, 20.5% (95% CI, 15.2%-26.3%; I^2, 91.88%; 25 studies); immigrants in general, 24.46% (95% CI, 15.23%-35.30%; I^2, 95.36%); blood donors, 12% (95% CI, 7%-18%; I^2, 0%); and pregnant women, 18.90%(95% CI, 12.76%-25.86%; I^2, 85.01%). There were no significant differences between the subgroups (p=0.145).

The prevalence was 17% (95% CI, 0%-49%) in immigrants from Argentina and 12% (95% CI, 0%-34%) in those from Paraguay. The prevalence was ≤2% for the remaining countries.

Cases where transmission is vertical, by transfusion, or by transplant

Twenty-seven cases of congenital infection were recorded from 502 live births (13 studies). The resulting pooled transmission prevalence was 3 per 100 live births (95% CI, 1-6 per 100 live births; I^2, 21.40%). Symptoms of infection were identified in 20% (95% CI, 0%-53%; I^2, 32.4%), and the most frequent sign was hepatosplenomegaly (5/7). All of the patients (100%) received treatment and progressed satisfactorily.

Mild-to-moderate organ involvement (presence of heart failure or LVEF <50%, ECG abnormality, or dysphagia/constipation)

<u>Cardiovascular symptoms</u>: The prevalence of cardiovascular symptoms (in general) was 43% (95% CI, 25%-63%; I^2, 95.45%), i.e., 302 patients in 1,448 cases (7 studies).

<u>Mild-to-moderate cardiovascular involvement</u>: The prevalence of mild-to-moderate cardiovascular disease was 19% (95% CI, 13%-27%; I^2, 88.36%), i.e., 343 cases in 1,946 seropositive patients (9 studies).

<u>Digestive symptoms:</u> The prevalence of symptoms such as dysphagia and constipation was 25% (95% CI, 18%-32%; I^2, 81.75%), i.e., 501 of 1,662 seropositive cases (9 studies).

<u>Mild-to-moderate gastrointestinal involvement:</u> The grouped prevalence of mild-to-moderate gastrointestinal involvement was 5% (95% CI, 2%-11%; I^2, 89.62%), i.e., 180 in 1862 seropositive patients (9 studies). The most frequently diagnosed gastrointestinal condition was dolichocolon, even though it is not specific to this disease.

Severe organ involvement (hospitalization due to heart failure, placement of a pacemaker, or diagnosis of megasyndrome)

There were 39 severe cardiac events in 1,946 patients (9 studies), i.e., a prevalence of 1% (95% CI, 0%-2%; I^2, 57.9%).

There were 45 cases of severe gastrointestinal involvement (megasyndrome) in 1,935 cases (11 studies), i.e., a grouped prevalence of 0% (95% CI, 0%-1%; I^2, 41.26%); in the worst case scenario, this could be up to 2.69% (95% CI, 1.89%-3.48%; I^2, 0%; 4 studies, uncorrected analysis).

Organ involvement (any type) after diagnosis

Organ involvement (any type) was recorded in 607 cases in 2,270 patients with Chagas disease, i.e., a grouped prevalence of 28% (95% CI, 18%-41%; I^2, 95.69%; 9 studies).

Indication for trypanocidal treatment

Fifteen studies report the treatment received, namely, benznidazole in all cases. This was indicated exclusively in 10 studies (67%), in which nifurtimox was also offered (5, 33%).

Trypanocidal treatment was started in 81% (95% CI, 67%-93%; I^2, 93.25%) of cases detected (1,062 patients, 1,790 cases, 11 studies).

Percentage who completed treatment

Of all the patients who started trypanocidal treatment, 78% (95% CI, 65%-89%; I^2, 88.5%) completed it (799 patients, 1,055 cases; 9 studies).

Adverse effects of treatment

Adverse effects were recorded in 47% (95% CI, 32%-63%; I^2, 90.45%) of patients who started trypanocidal treatment (638 of 996 cases, 8 studies).

Quality of life

No outcomes were reported in any of the studies selected.

Mortality

Two patients died suddenly of a cardiac condition secondary to Chagas disease.

3. REVIEW FINDINGS

3.1. Results of the search

The search yielded 470 references. Based on the eligibility criteria, we identified 65 references for which the complete text was reviewed. Of these, we selected 33 studies, all of which fulfilled the inclusion criteria. A PRISMA flow diagram details the eligibility process.

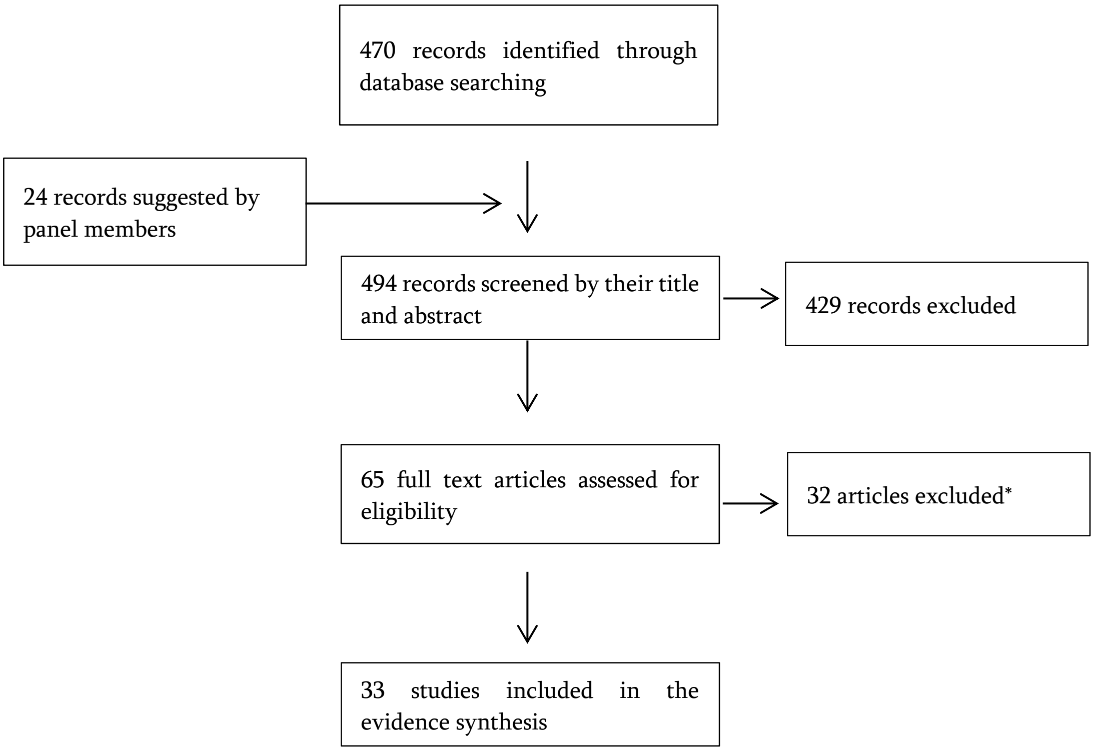

STUDY ELIGIBILITY. PRISMA FLOW DIAGRAM **Reason for exclusion of full-text articles: 12 narrative review articles; 9 conference abstracts; 4 studies in endemic settings; 2 cost studies; 2 duplicates; 1 case report; 1 study in a non-eligible setting; 1 registry report.*

3.2. Characteristics of the studies included

Most of the studies selected were performed in Spain (23, 69.70%); the rest were from France (El Ghouzzi 2010, Lescure 2008, Lescure 2009), Switzerland (Jackson 2010, Martinez 2009), Italy (Gabrielli 2013, Angheben 2011), Canada (O'brien 2013), EuroTravNet (Perez-Molina 2011), and Germany (Frank 1997).

The studies were observational, and most collected the information prospectively (20; 58.82%). Five were retrospective, 8 cross-sectional, and 1 was a case series (Lescure 2008). The study period varied, with the shortest being 4 months (Frank 1997) and the longest 148 months (Angheben 2011).

Of the 35 different populations described in the 33 studies (1 study reported on 3 populations with different characteristics [Angheben 2011]), the type of population and circumstances of screening made it possible to differentiate between 3 contexts in which the studies were performed.

1) Studies in ***immigrants*** in general seen at outpatient centers (16, 46%): most were in symptomatic or asymptomatic immigrants who attended general medical outpatient clinics, primary care centers, or centers specialized in tropical and infectious diseases. The strategies for recruiting patients were

heterogeneous, with some studies including community activities and health promotion activities in populations at risk, whereas others depended on opportunistic screening of all patients from endemic areas who attended the clinic for any reason.

2) Studies in ***blood donors*** performed in blood banks (6, 17%) to evaluate programs for screening of *T. cruzi* infection in the process of being implemented or already implemented.

3) Studies in ***pregnant women*** within antenatal screening programs (13, 37%) that had been implemented or were in the process of implementation in hospitals, with the participation of primary care centers. These were generally carried out during the first trimester or at delivery, with follow-up of the newborns of seropositive pregnant women. One study did not present results for the follow-up of seropositive mothers in newborns (Soriano 2009).

Several screening tests were used, although these generally involved the combination of an ELISA-type test with an indirect immunofluorescence technique and other confirmatory tests that varied depending on the individual study. The criteria for a positive result generally fulfilled WHO recommendations, according to which chronic infection by *T. cruzi* is defined as any asymptomatic patient who has 2 positive results in separate serology tests.
Appendices III to V present the detailed characteristics of the studies selected according to the clinical context in which they were performed.

3.3 Trypanosomiasis: disease burden in immigrants

Impact at international level and in Europe

Chagas disease (American trypanosomiasis) is caused by the parasite *T. cruzi*, which was first described by the Brazilian doctor Carlos Chagas in 1909. It is estimated that 8-10 million persons throughout the world are chronically infected, 50,000 new cases are reported every year, and approximately 28 million people are at risk of becoming infected. The disease is endemic in 21 countries in Latin America[1], where it is mainly transmitted by vectors. According to data from 2010, 5,742,167 people were infected by *T. cruzi* in 21 Latin American countries. Of these, 62.4% were in the Southern Cone. Argentina, Brazil, and Mexico were home to the largest number of persons infected, followed by Bolivia. In the Andean region, the number of people infected reached 958,453. Of these, 45.7% (437,960) were from Colombia. The countries with 100,000–200,000 infected persons include 2 from the Southern Cone (Chile and Paraguay), 3 from the Andean region (Venezuela, Ecuador, Peru), and 1 from Central America (Guatemala) (WHO 2015).

Chagas disease continues to be the leading cause of myocardial heart disease in Latin America. The WHO classified it, along with other infectious tropical diseases, as a neglected disease (WHO 2016).

In Europe, Chagas disease is not monitored systematically, although available data suggest that prevalence rates are fairly high in some countries. The largest numbers of cases in Europe are found in Spain, Italy, the Netherlands, the United Kingdom, Germany, and France (ECDC 2014).

According to the study by Basile et al (Basile 2011), in 2009, a total of 4,290 cases of Chagas disease were diagnosed in Latin American immigrants in 9 European countries, with a prevalence of 1.3 per 1,000 migrants from endemic countries. The prevalence among undocumented migrants may be even greater. The estimated number of cases of congenital Chagas disease ranges from 20 to 183 in the countries in question.

Spain

Spain is home to the largest number of persons from Latin America outside the USA and has the highest number of confirmed cases of Chagas disease in Europe. Prevalence differs for each group of immigrants according to the country and the specific area of origin.

---

(1) Argentina, Belize, Bolivia (Plurinational State of), Brazil, Chile, Colombia, Costa Rica, Ecuador, El Salvador, French Guiana, Guatemala, Guyana, Honduras, Mexico, Nicaragua, Panama, Paraguay, Peru, Suriname, and Venezuela (Bolivarian Republic of).

According to a report on Chagas disease and blood donation from the Ministry of Health in 2009 based on the immigrant population, it was estimated that there were 53,000 possible carriers in Spain and 0.02-2.35 infected donations per 1,000. Considerable heterogeneity was detected between Autonomous Communities (Enfermedad de Chagas y donación de sangre. Ministerio de Sanidad 2009).

According to an estimation based on the number of immigrants from Latin America in the 2011 census and after applying values from the study by Basile et al (Basile et al 2011) for each of the countries of origin, in Spain, 68,636 Latin American immigrants are infected by *T. cruzi*. Of these, 22,100 are thought to be women of childbearing potential (Imaz 2015).

In line with the recommendations of the latest European technical report on infectious diseases in immigrants, the key issues to resolve in the treatment of Chagas disease are prevention of transmission via donation of blood, organs, tissues, and cells by people from Latin America and congenital transmission in pregnant Latin American women infected by *T. cruzi* (ECDC 2014).

3.4. REVIEW FINDINGS FOR THE OUTCOMES OF INTEREST

3.4.1 Patients with infection: prevalence of Chagas disease

We found 1,441 cases in a population of 19,735 immigrants from endemic areas. This outcome was reported in 28 studies.

The total pooled prevalence of infection by *T. cruzi* was 6.08% (95% CI, 3.24%-9.69%; I^2, 98.82%). From the total number of cases where it was possible to identify the country of origin (986 cases, 17 countries), 90% were from Bolivia, followed by Argentina (32, 3.3%), Paraguay (25, 2.5%), and Ecuador (12, 1.22%).

Significant differences were found between the subgroups by subtype of study according to the context in which screening was performed, as follows:

a. **Immigrants in general:** Studies performed at community level or in outpatient clinics in a population that had previously been identified as being at risk and that could or could not have presented with symptoms associated or not with infection. In this type of study, screening for Chagas disease was performed on immigrants from endemic countries. In this context, the prevalence was 13.30% (95% CI, 7.30%-20.71%; I^2, 98.15%), i.e., 1,000 from a total of 5,826 immigrants (12 studies).

b. **Pregnant women:** Studies performed as part of a screening strategy that was being implemented or had already been implemented as part of an antenatal or perinatal care program aimed at pregnant women from endemic areas mainly in the Southern Cone. The prevalence in this case was 4% (95% CI, 2%-7%; I^2, 96.28%), with 393 cases from 6,407 screened pregnant women (10 studies).

c. **Blood donors:** Studies that describe screening that was specific for Chagas disease in previously established programs in blood/blood derivative donation centers. The studies selected were those in which it was possible to identify a denominator. In this case, the grouped prevalence was 0.42% (95% CI, 0.03%-1.08%; I^2, 82.06%), with 48 cases in 7,502 persons screened (6 studies).

Figure 1. Prevalence of *Trypanosoma cruzi* infection in immigrants

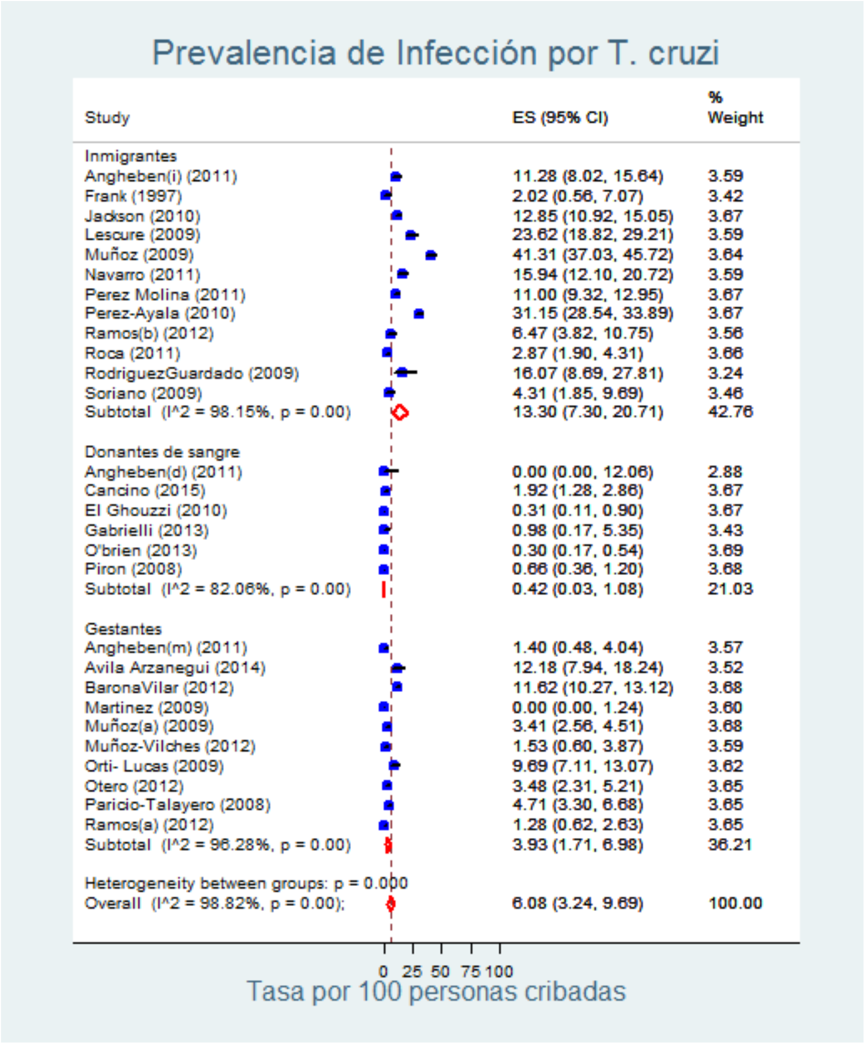

Prevalence by country of origin

In ***persons from Bolivia***, the global prevalence of *T. cruzi* infection was 20.51% (95% CI, 15.23%-26.3%; I^2, 91.88%; 25 studies). By area of study, at community level, the prevalence was 24.46% (95% CI, 15.23%-35.30%; I^2, 95.36%) among immigrants in general, 11.78% (95% CI, 6.74%-17.7%; I^2, 0%) in blood donors, and 18.90%(95% CI, 12.76%-25.86%; I^2, 85.01%) in pregnant women, with no significant differences between subgroups (p=0.145) (Figure 2).

Figure 2. Prevalence of *Trypanosoma cruzi* in persons from Bolivia

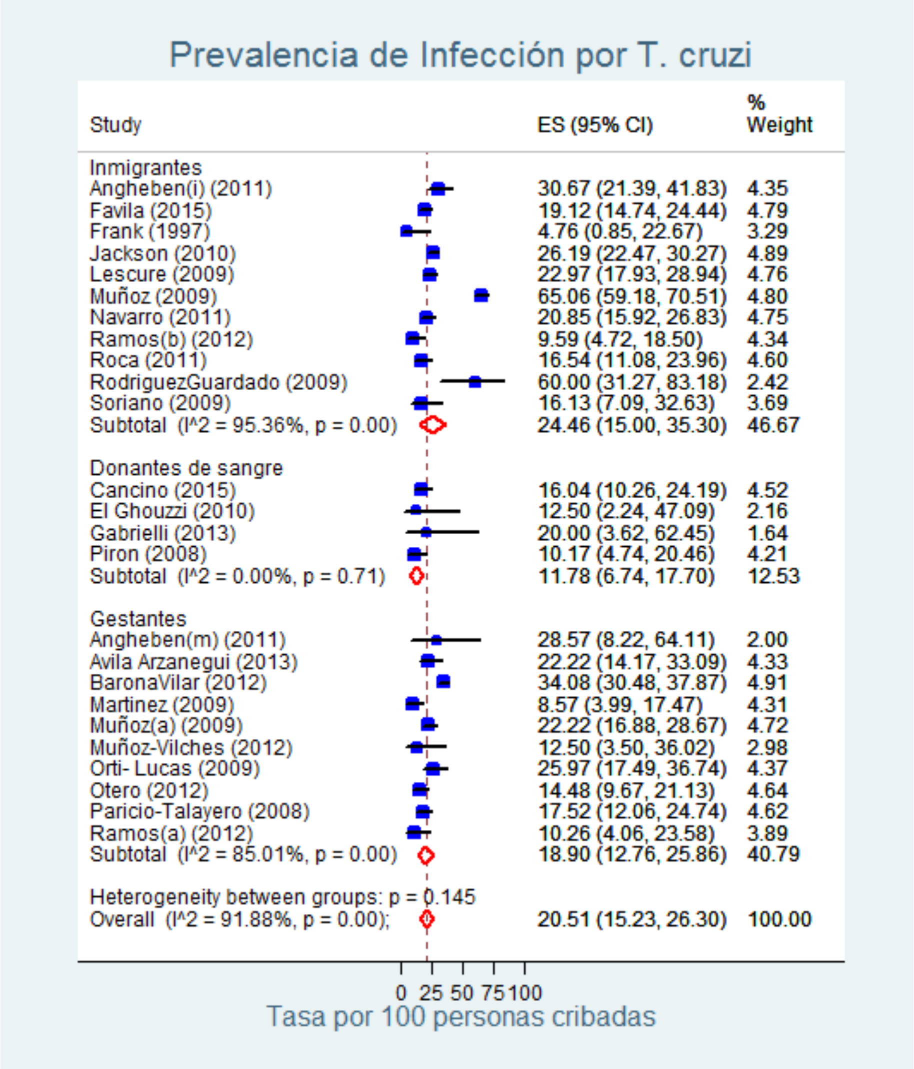

Grouped prevalence in immigrants (community level or outpatient clinics) reached 17% (95% CI, 0%-49%) in those from Argentina and 12% (95% CI, 0%-34%) in those from Paraguay. In the remaining countries, the grouped prevalence was ≤ 2%, although broad confidence intervals were observed in countries such as Honduras, Chile, Mexico, Costa Rica, Panama, and Guatemala, probably owing to the low number of participants from these countries. No cases were detected in immigrants from 5 countries who did in fact undergo screening (Costa Rica, Panama, Uruguay, Guatemala, and Guyana) (Data by country in Appendix VI.

The heterogeneity index (I^2) was high in all subgroups, both in the overall analysis and in the analyses of Bolivians. The differences between subgroups can probably be explained by the characteristics of the patients who attended the different health centers and the differences in the strategies used to recruit patients.

3.4.2 Cases of vertical transmission and transmission by transfusion or transplant

Vertical transmission

A total of 27 cases of infection by vertical transmission were collected from 13 studies covering 502 live births in mothers with chronic *T. cruzi* infection. The resulting rate of grouped transmission is 3 per 100 live births (95% CI, 1-6 per 100 live births; I^2, 21.40%).

Figure 3. Rate of vertical transmission of *Trypanosoma cruzi* infection

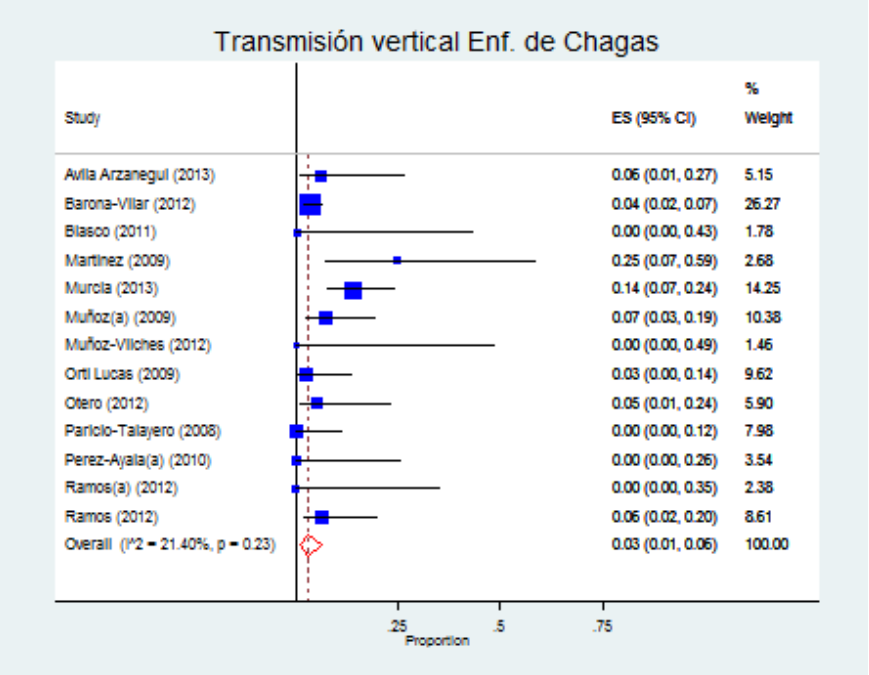

Cases of Chagas disease by vertical transmission

Of the 13 studies, 12 were carried out in Spain and 1 in Switzerland (Martínez 2009). With respect to timing, most pregnant women underwent screening during the first trimester of pregnancy. The grouped prevalence of infection in pregnant women in the studies with follow-up data (9 studies) was 5% (95% CI, 2%-8%). Bolivia was the country of origin for 88% of the pregnant women with chronic *T. cruzi* infection (95% CI, 78%-96%; I^2, 79.65%), with a mean (SD) age (reported in 2 studies) of 28.55 (5.7) years (Appendix VII).

Figure 4. Prevalence of *Trypanosoma cruzi* infection in studies of pregnant women

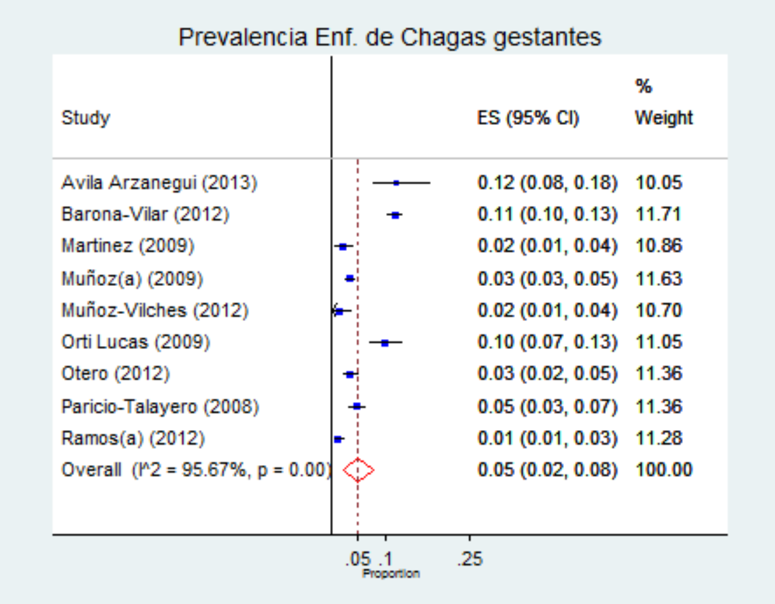

In cases of neonatal infection where the country of origin of the mother was provided, 100% were Bolivian. Of the 27 cases of neonatal infection, 7 had symptoms associated with *T. cruzi* infection (20%; 95% CI, 0%-53%; I^2; 32.4%). The most frequent signs and symptoms included hepatosplenomegaly (5/7), low birth weight (4/7), prematurity (3/7), cholestasis (1/7), heart failure (2/7), cholestasis (1/7), and pulmonary hypertension (1/7) (Appendix VII).

Outcome was satisfactory in 100% of cases, and symptoms resolved with treatment. There were no deaths.

Figure 5. Prevalence of symptoms in neonates infected by *Trypanosoma cruzi*

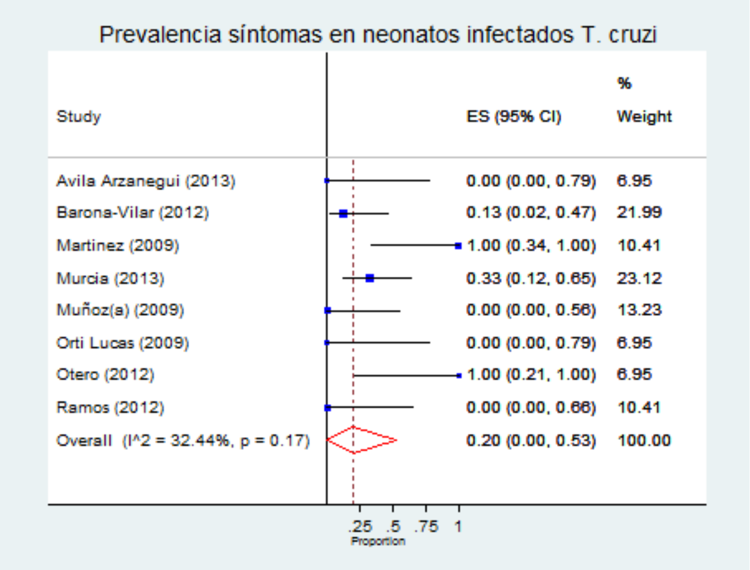

Transmission by transfusion or transplant

There were no cases of transmission by transfusion or transplant in the studies selected.

The literature provides documented evidence of cases of Chagas disease diagnosed in people with no history of travel to endemic areas or of previous infection who had received a solid organ transplant (heart, liver, kidney), bone marrow transplant, or blood packs and/or blood derivatives via transfusion in whom infection by *T. cruzi* was investigated retrospectively. Most evidence in these cases is from endemic countries (Martin Dávila 2008, Cantarovich 1992, D'Albuquerque 2007, De Paula 2008).

In non-endemic countries, there were also cases where Chagas disease was transmitted by transfusion of blood products or by transplant (Martin Dávila 2008). In Spain, there have been isolated cases secondary to infection caused by bone marrow transplant and/or use of infected blood products from donors from Latin America (Villalba 1992, Forés 2007, Flores-Chávez 2008).

3.4.3. Mild-to-moderate organ involvement (presence of heart failure or LVEF <50%, ECG abnormalities, or dysphagia/constipation)

Neonates

In neonates infected by their mother, there were 7/27 patients who presented symptoms such as abdominal distension with hepatosplenomegaly (5/7), low birth weight (4/7), and prematurity (5/7).

Persons aged >14 years

Twenty of the 34 studies report symptoms in seropositive cases, albeit with wide variability in the method of reporting them, which ranges from stating whether the patient is symptomatic or asymptomatic to specification of the clinical stage according to current classifications, for example, that of the modified Brazilian Consensus (2 studies, Valerio 2012 and Jackson 2010), the Kuschnir classification (Salvador 2013) for heart failure, or according to electrocardiographic and echocardiographic criteria as defined in the individual study (e.g. Gascón in Muñoz et al. 2009). In each study, the presence of symptoms may only involve the mention of their absence or, in contrast, a detailed description of the symptoms. Symptoms in pregnant women are described in section 3.4.2 and in Appendix VII. Fifty cases were found in immigrants screened in blood banks; these studies do not report the symptoms in these cases, except for a study by Gabrielli et al. in 2013, where a positive patient was asymptomatic (Appendix VIII).
The meta-analysis was performed taking into account only studies among immigrants, because they probably better reflect what really occurs in the community (Appendix IX).
The classification of gastrointestinal involvement was not very specific or was based on international classifications such as that of Rezende for esophageal achalasia or standard classifications such as considering colon diameter greater than 6 cm as pathological. The standard assessments included esophagography or barium enema in patients with gastrointestinal symptoms.

Heart involvement

Cardiovascular symptoms

In studies carried out in immigrants in general, the grouped prevalence of cardiovascular symptoms (e.g., dyspnea, palpitations, precordial pain, edema) in the cases detected was 43% (95% CI, 25%-63%; I^2, 95.45%), with considerable heterogeneity between the studies: 302 had these symptoms at the time of screening (1,448 cases, 7 studies) (Appendix IX).

Figure 6: Prevalence of cardiovascular symptoms in immigrants with Chagas disease

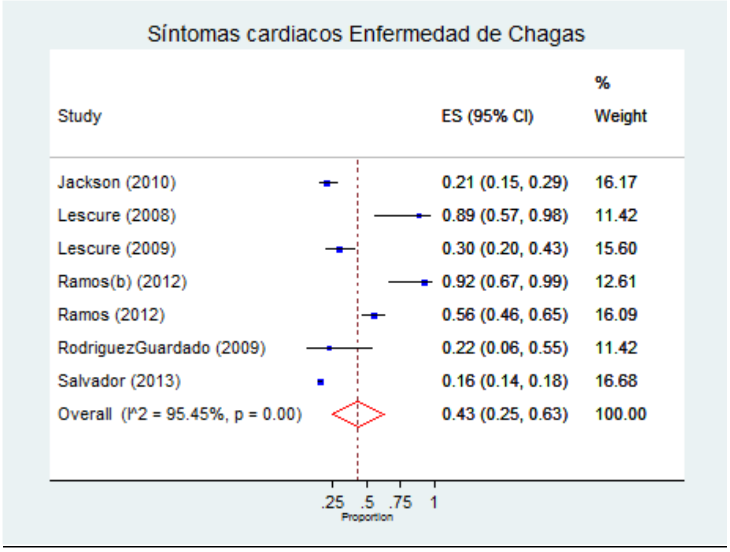

Mild-to-moderate heart involvement

The prevalence of mild-to-moderate heart involvement in immigrants in general was 19% (95% CI, 13%-27%; I^2, 88.36%); in 343 cases, evidence of cardiac abnormalities was provided by electrocardiographic tests and other techniques (1,946 seropositive patients, 9 studies) (Appendix IX).

Figure 7. Prevalence of mild-to-moderate cardiac involvement in patients with Chagas disease

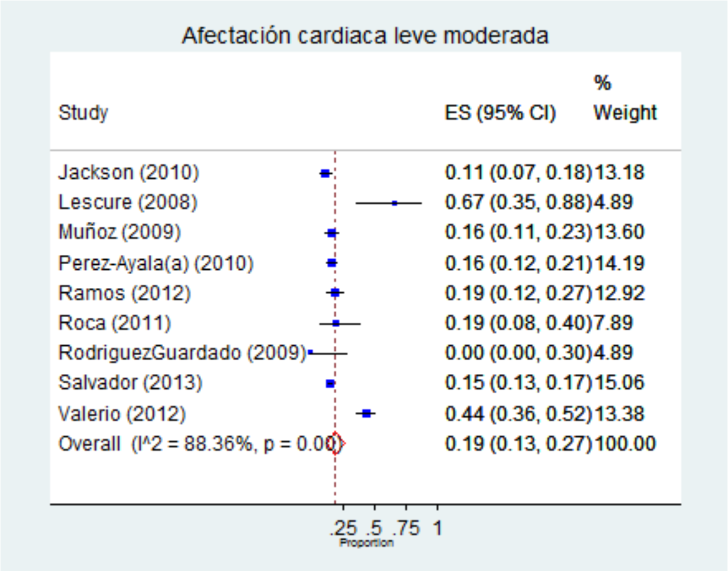

Gastrointestinal involvement

Gastrointestinal symptoms

The grouped prevalence of gastrointestinal symptoms at the time of screening in cases with Chagas disease was 25% (95% CI, 18%-32%; I^2, 81.75%). We recorded 501 cases with symptoms of gastrointestinal involvement such as dysphagia, constipation, and other symptoms in immigrants in general (1,662 patients who were seropositive for *T. cruzi*, 9 studies) (Appendix IX).

Figure 8. Prevalence of gastrointestinal symptoms in patients with Chagas disease

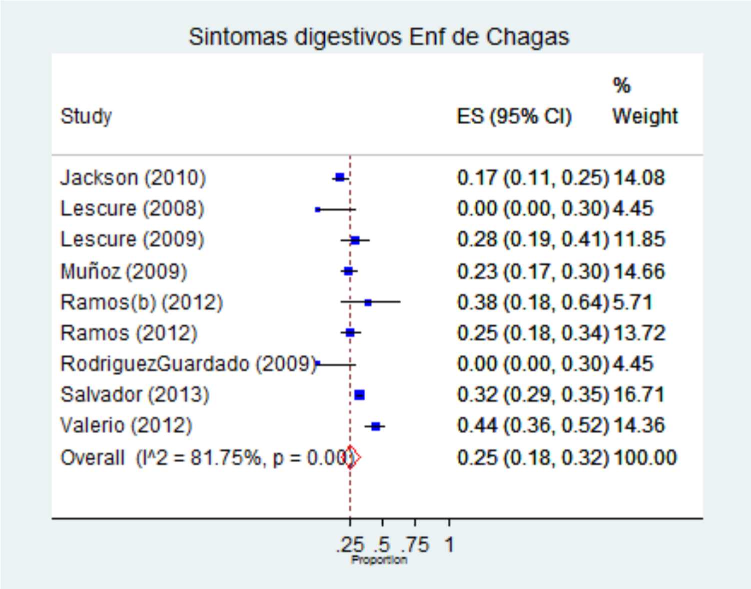

Mild-to-moderate gastrointestinal involvement

Studies assessing the disease showed that the prevalence of gastrointestinal involvement was 5% (95% CI, 2-11%; I^2, 89.62%), with 180 cases in 1,862 seropositive patients (9 studies). The most common diagnosis was dolichocolon, even though this was not specific to Chagas disease (Appendix IX).

Figure 9. Prevalence of mild-to-moderate gastrointestinal involvement in Chagas disease

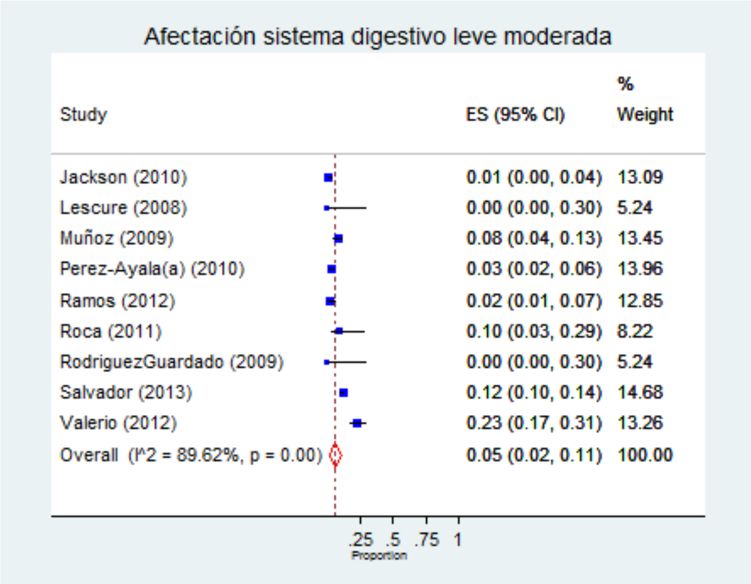

3.4.4 Severe organ involvement (hospitalization for heart failure, need for a pacemaker, or diagnosis of megasyndrome)

Neonates

In the case of neonates with symptomatic *T. cruzi* infection, 2 had heart failure during the course of the disease and 1 experienced complications in the form of respiratory insufficiency caused by severe pulmonary hypertension and hepatosplenomegaly with cholestasis.

Persons aged >14 years

Severe cardiac involvement

A total of 39 cases of severe cardiac events were recorded in 1,946 patients (9 studies) with a prevalence of 1% (95% CI, 0%-2%; I^2, 57.9%). The most frequent severe heart conditions were rhythm disorders, severe block requiring placement of a pacemaker, severe heart failure, Chagas heart disease while waiting for a transplant, stroke, and other conditions. In addition, 2 patients died owing to heart-related complications (Muñoz 2009 and Pérez-Ayala 2010) (Appendix IX).

Figure 10. Prevalence of severe cardiac involvement in patients with Chagas disease

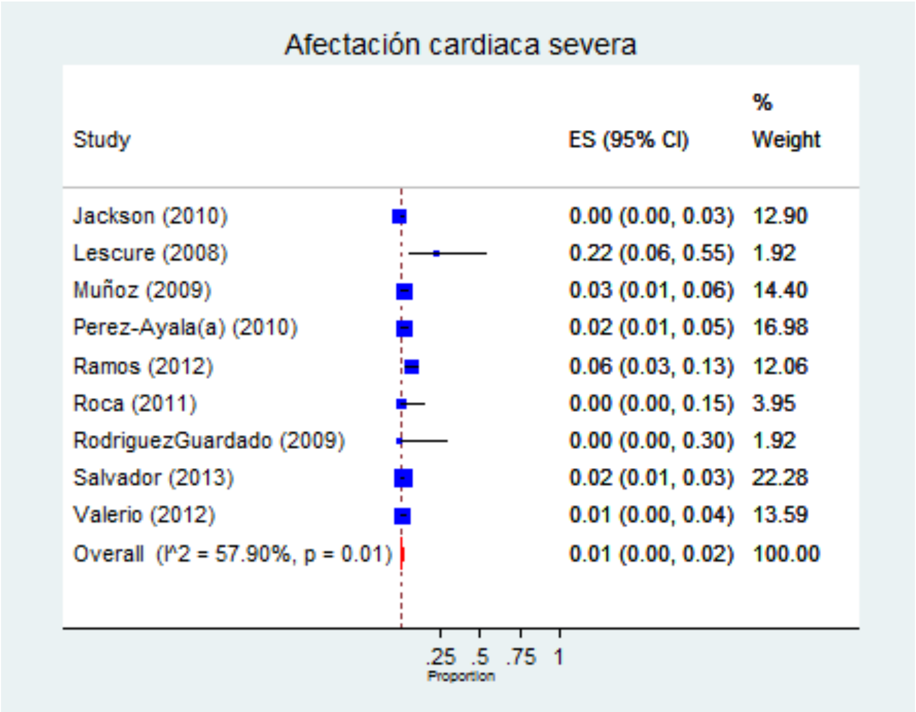

Digestive tract involvement: megasyndrome.

A total of 45 cases of severe gastrointestinal involvement were collected from 1,935 cases (11 studies) in immigrants. The grouped prevalence was between 0% and 1% (I^2, 41.26%). An uncorrected analysis (excluding studies with no observed cases of megasyndrome) revealed the grouped prevalence to be 2.69% (95% CI, 1.89%-3.48%; I^2, 0%; 4 studies). The main diagnoses were megasyndrome affecting the colon, esophagus, and stomach (Appendix IX).

Figure 11. Prevalence of severe gastrointestinal involvement: megasyndrome

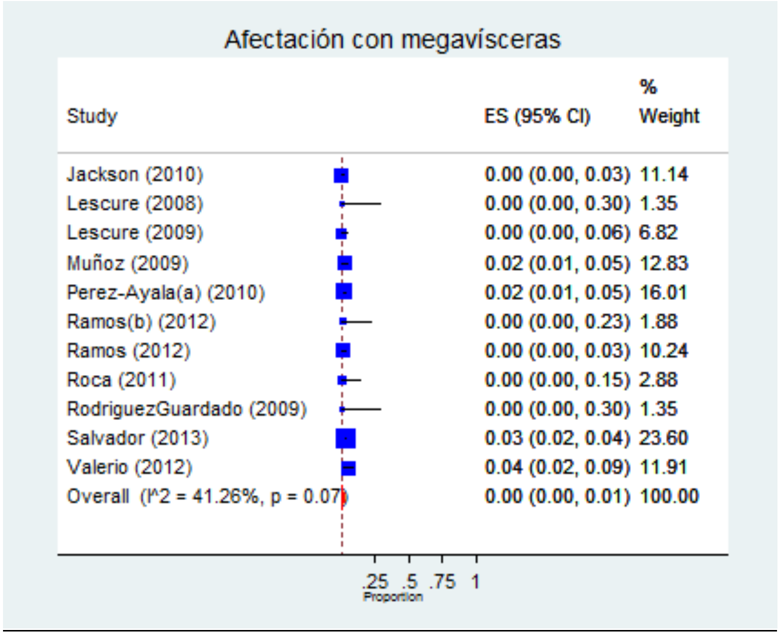

3.4.5 Organ involvement (any type) after diagnosis

The prevalence of organ involvement (any degree of severity) in patients diagnosed with Chagas disease was 28% (95% CI, 18%-41%; I^2, 95.69%; 607 patients with involvement from 2,270 cases; 9 studies).

Figure 12. Prevalence of organ involvement (any type) in patients with Chagas disease

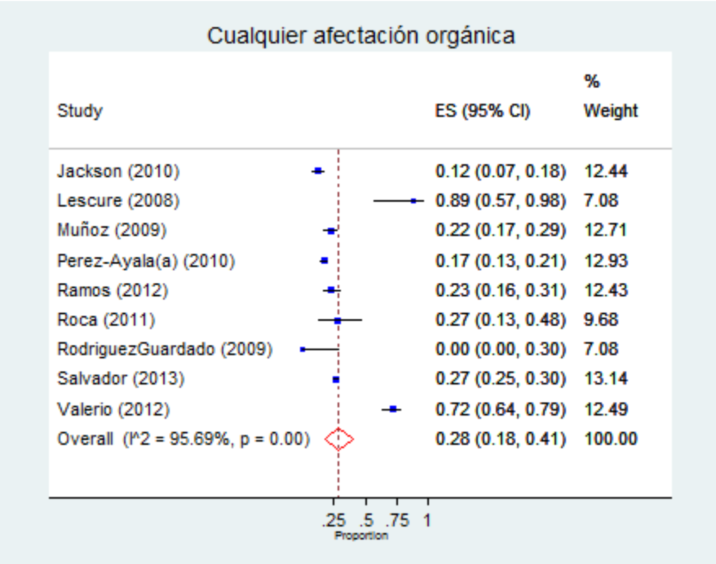

3.4.6 Treatment received: Indication for trypanocidal treatment

The outcome of trypanocidal treatment is reported in 42% (15/36) of studies. The most commonly prescribed drug was benznidazole (10, 67%); in other studies, nifurtimox was offered as an alternative to benznidazole or retained as second-line therapy (5, 33%) (Salvador 2013, Valerio 2012, Lescure 2008, Martínez 2009, Pérez-Ayala (a) 2010). Trypanocidal therapy was started by 81% (95% CI, 67%-93%; I^2, 93.25%) of seropositive

adults (1,062 of 1,790 cases; 11 studies), with no significant differences between immigrants or in pregnant women (p=0.721) (Appendices X - XI).

Figure 13. Patients who started treatment for Chagas disease

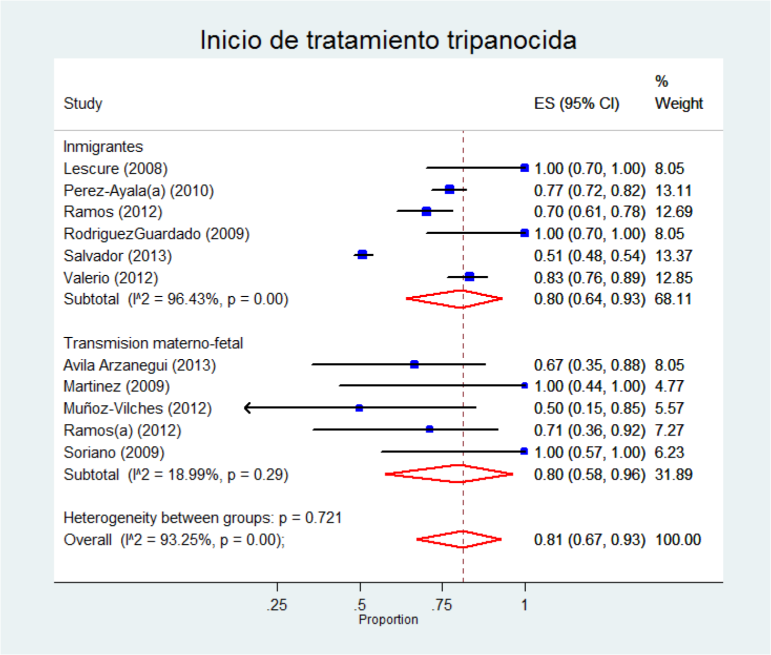

3.4.7 Percentage of patients who completed treatment

Of all the patients who began trypanocidal treatment, 78% (95% CI, 65%-89%; I^2, 88.5%) completed the treatment course (799 of 1,055 cases, 9 studies). The reasons for discontinuation were mild or severe adverse effects (Appendix X).

Figure 14. Percentage of patients who completed treatment

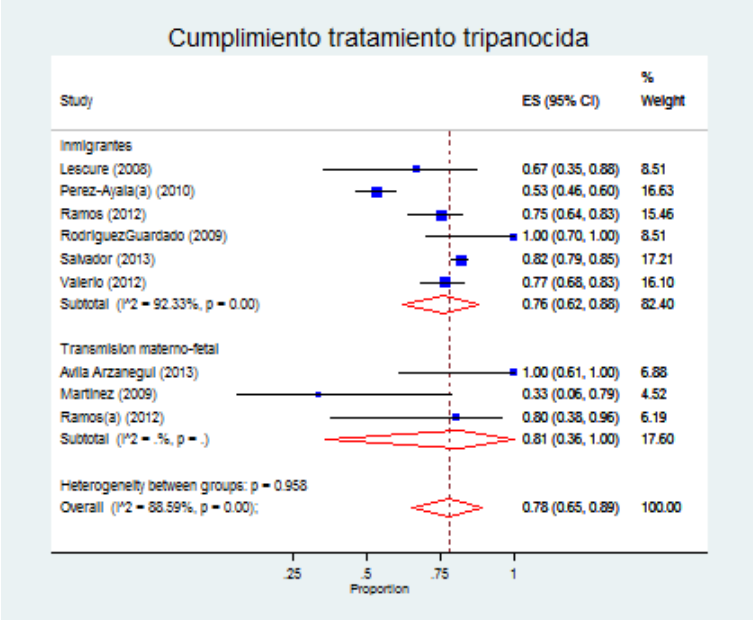

The usefulness of trypanocidal treatment in adults with Chagas disease is somewhat questionable. A meta-analysis from the year 2009 showed a limited benefit for treatment of chronic disease with benznidazole, with a marginal effect compared with placebo (Perez Molina 2009). The only randomized clinical trial comparing benznidazole with placebo did not show any benefit for this drug in terms of clinical outcomes (development of heart disease or death), and only patients treated with benznidazole continued to have undetectable *T. cruzi* in PCR more than those who received placebo, although this was not associated with clinical outcomes (Morillo 2015). In this study, the participants had moderate-to-severe heart disease; therefore, in patients with no organ involvement or with mild involvement, we do not know whether benznidazole could in fact control the disease. Furthermore, recent clinical trials on the use of imidazoles (posaconazole and ravuconazole) in monotherapy (Morillo 2017) or in combination with benznidazole (Morillo 2017, Torrico 2018) have proven less efficacious than benznidazole in terms of microbiological markers of response (PCR for *T. cruzi*).

3.4.8 Treatment-related adverse effects (any type)

Of 996 patients with Chagas disease who started trypanocidal treatment, 638 experienced an associated adverse event, that is, a prevalence of 47% (95% CI, 32%-63%; I^2, 90.45%; 8 studies), with no differences according to the context of the study (p=0.6). The adverse effects reported included drug-related eosinophilia with systemic syndrome (Lescure 2008), skin rash, peripheral neuropathy, leukopenia, taste disorders, gastrointestinal disorders, and headache (Appendix X).

Figure 15. Prevalence of adverse effects associated with trypanocidal treatment

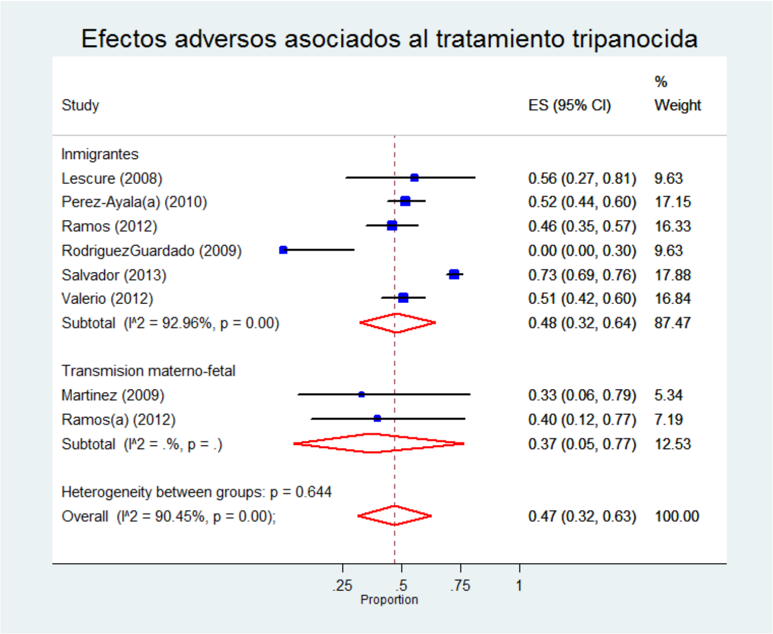

Given the lack of quality studies, the usefulness of treatment in the asymptomatic phase is questionable. The efficacy of treatment in chronic infection may be questionable. The marginal effect of treatment in this stage of the disease could result from the major differences between studies (all non-randomized) (Perez Molina 2009). Furthermore, a systematic review focusing on the safety of treatment has shown that benznidazole is poorly tolerated, with a 44% frequency of adverse effects, together with a considerable dropout rate (11%) (Crespillo-Andújar 2018).

3.4.9 Quality of life

No reference is made to quality of life in the studies selected.

3.4.10 Mortality

The studies selected recorded patients with chronic *T. cruzi* infection who died of severe Chagas cardiopathy (Muñoz 2009, Pérez-Ayala 2010).

4. Appendices

APPENDIX I - SEARCH STRATEGY DESIGNED TO OBTAIN RELEVANT STUDIES FOR THE REVIEW

MEDLINE (PubMed) Oct 2018

#1	"Emigration and Immigration"[Mesh]	24604
#2	"Emigrants and Immigrants"[Mesh]	10947
#3	"Transients and Migrants"[Mesh]	10650
#4	"Refugees"[Mesh]	9203
#5	immigr*[tiab]	30582
#6	emigr*[tiab]	7422
#7	migrat*[tiab]	283296
#8	migrant*[tiab]	17294
#9	diaspor*[tiab]	704
#10	refugee*[tiab]	9102
#11	asylum seek*[tiab]	1419
#12	#1 OR #2 OR #3 OR #4 OR #5 OR #6 OR #7 OR #8 OR #9 OR #10 OR #11	341580
#13	"Early Diagnosis"[Mesh]	43425
#14	"Mass Screening"[Mesh]	120866
#15	screen*[tiab]	664860
#16	tested[tiab]	874658
#17	testing[tiab]	488277
#18	prevention and control[tiab]	18782
#19	preventi*[ti]	201160
#20	earl[ti]	169
#21	early[ti]	267294
#22	#13 OR #14 OR #15 OR #16 OR #17 OR #18 OR #19 OR #20 OR #21	2340870
#23	"Trypanosoma cruzi"[Mesh]	11174
#24	Trypanosoma cruzi[tiab]	13099
#25	T. cruzi[tiab]	7962
#26	#23 OR #24 OR #25	14811
#27	#12 AND #22 AND #26	169

OVID Embase 1974 to 2018 October

1 exp migration/ (42837)
2 exp migrant/ (33552)
3 exp refugee/ (11890)
4 (immigr* or emigr* or migrat* or migrant* or diaspor* or refugee* or asylum seek*).ti,ab. (417669)
5 1 or 2 or 3 or 4 (436401)
6 exp early diagnosis/ (100134)
7 exp mass screening/ (225168)
8 (screen* or tested or testing).ti,ab. (2548829)
9 (preventi* or earl or early).ti. (588436)
10 6 or 7 or 8 or 9 (3195672)
11 exp Trypanosoma cruzi/ (14808)
12 (Trypanosoma cruzi or T cruzi).ti,ab. (15236)
13 11 or 12 (17656)
14 5 and 10 and 13 (301)

APPENDIX II. CHARACTERISTICS OF THE STUDIES INCLUDED

Characteristic	n	%	
**Place (n=33)**			
Spain	23	69.70%	
France	3	9.09%	
Switzerland	2	6.06%	
Italy	2	6.06%	
Canada	1	3.03%	
Germany	1	3.03%	
EuroTravNet	1	3.03%	
**Design (based on data collection method)**			
Prospective	20	60.61%	
Retrospective	4	12.12%	
Case series	1	3.03%	
Cross-sectional	8	24.24%	
**Scope of the study (n=35)**[1]			
Immigrants	16	46%	
Mother-to-child l transmission	13	37%	
Blood donors	6	17%	
**Observation period based on type of study (months) (n=32)**[2]	Mean	Min	Max
Prospective	32.3	12	72
Retrospective	87.67	13	148
Case series (1)	24		
Cross-sectional	20.625	4	60
Total	33	100%	

[1]One study reported results from 3 subpopulations in different contexts (Angheben 2011).

[2]One study did not specify the period (Blasco 2011).

APPENDIX III. STUDIES IN IMMIGRANTS SEEN AT OUTPATIENT CENTERS

Author, year, country	Setting	Type of study	Objectives	Dates of study, months of follow-up	Screening test	Definition of a positive result
Angheben 2011(i) Italy	2 centers providing care to immigrants, and a screening program in an at-risk population	Retrospective	To estimate the prevalence of *T. cruzi* infection in Latin American immigrants	April 1998– April 2010, 148 months	Immunochromatography assay (Chagas Quick Test, Cypress Diagnostics, Belgium)<br>BioELISA Chagas, Biokit S.A., Spain or *Trypanosoma cruzi* lysate (DRG CHAGAS IgG, Germany).<br>Two ELISA tests in some cases.	In the case of discordant results, a third test was performed according to the recommendations of the WHO
Favila 2015 Spain	Primary care center in Mallorca (Spain)	Cross-sectional	To estimate the prevalence of Chagas disease in Bolivian patients selected via systematic sampling.	October 2011-March 2012, 7 months	Immunochromatography assay (SD Chagas Rapid Bioline Standard Diagnostics, Inc), sensitivity 90.4% and specificity 94%. ELISA (in-house IFI), sensitivity 98.5% and specificity 100%	NR
Frank 1997 Germany	Latin American support groups (Caritas)	Cross-sectional	To evaluate the presence and epidemiological impact of *T. cruzi* infection in Latin American immigrants in Germany.	May to August 1995, 4 months	Indirect immunofluorescence assay (IIF with epimastigotes) (Bits/Germany), ELISA (Institute of Tropical Medicine, Berlin).	Samples that were seroreactive to antibodies for *T. cruzi* in IIF and ELISA and did not react with the Leishmania antigen were considered positive.
Jackson 2010 Switzerland	Primary care center attending undocumented immigrants in 2 parishes	Cross-sectional	To determine the prevalence of Chagas disease among Latin American immigrants. To evaluate risk factors, clinical stage, and transfusion and transplant risk in the local population.	June - December 2008, 6 months	Serologic ELISA-test (ELISA cruzi, bioMérieux, Brazil and Bioelisa Chagas, Biokit, Spain)	A diagnosis of Chagas disease was confirmed when the results of both tests were positive.
Lescure 2008 France	Hospital	Case series	Description of 9 cases in France.	2004-2006<br>24 months	Indirect immunofluorescence; ELISA BM, Biomérieux; BK, Biokit;	NR
Lescure 2009 France	Advertising campaign in the Latin American community	Prospective	To evaluate interest in selective screening in the population at risk in France	June 2008 -June 2009<br>13 months	Immunofluorescence, Elisa Biokit®, Elisa bioMérieux ® and Elisa Wiener®	NR
Muñoz 2009 Spain	Centers specialized in infectious diseases	Prospective	To describe the clinical profile of a series of Latin American patients at risk	July 2004-July 2007<br>37 months	BioELISA Chagas®, in-house Biokit S.A. ELISA with complete *T. cruzi* epimastigote antigens.<br>Western blot, with *T. cruzi* epimastigote antigens.<br>Nested PCR with blood and real-time PCR	Infected if seropositive in 2 serology tests. Nested PCR with blood (Marcon et al., 2002) and real-time PCR (Pirón et al., 2007) for seropositive patients.
Navarro 2011 Spain	Community, tropical diseases unit, Ramón y Cajal Hospital Program aimed at immigrants from Bolivia and Latin American women of reproductive age	Prospective	To describe a holistic care program for the management of Chagas disease: "Nuevos ciudadanos nuevos pacientes" (New Citizens, New Patients).	2008 – 2009<br>12 months	Rapid immunochromatography assay (ICT) (Simple Chagas WB, Operon), blood sample from finger prick, indirect fluorescent antibodies, and immunoenzyme assay	NR
Pérez Molina 2011 EuroTravNet	Clinics for travelers and immigrants: outpatient clinic.	Prospective	To describe the characteristics of patients diagnosed with Chagas disease in EuroTravNet, a network for tropical and travel medicine.	1 January 2008-31 December 2009<br>24 months	Chronic cases: IgG antigens *T. cruzi* ELISA, IFI, or indirect hemagglutination.<br><br>Acute cases, direct method: microhematocrit, Strout test, or Giemsa stain.	Chronic: Two positive serology tests<br>Acute: If positive result in any direct method.
Pérez-Ayala(a) 2011 Spain	Hospital: tropical diseases unit	Prospective	To describe the clinical-epidemiological characteristics of Latin American immigrants with	2003-2009<br>72 months	ELISA and IFAT PCR<br>ECG and echocardiogram (ECC)	NR

Author, year, country	Setting	Type of study	Objectives	Dates of study, months of follow-up	Screening test	Definition of a positive result
			chronic *T. cruzi* infection and provide preliminary data on treatment with benznidazole.		Esophageal manometry, barium meal, and barium enema only in symptomatic patients	
Ramos(b) 2012 Spain	Recruitment of patients through community activities in associations of immigrants from Paraguay and Bolivia who live in Elche	Cross-sectional	To investigate the prevalence of Chagas disease	November 2009-November 2010, 12 months	Capillary samples: Whatman protein saver 903 card (Whatman GmbH, Dassel, Germany)<br>IgG anti-*T. cruzi*<br>ELISA, IFAT	Must have 2 positive serology tests. PCR 121–122 and Tcz1–Tcz2 was requested
Ramos 2012 Spain	Hospital. Four hospitals in the province of Alicante using a similar protocol	Retrospective	To describe our experience in the treatment of Chagas disease	January 2002-May 2011, 102 months	ELISA or immunochromatography assay, (Novagnost Chagas IgG, NovaTec Immunodiagnostica GmbH, Dietzenbach, Germany; BioElisa Chagas Biokit, Biokit Barcelona; Chagas Stat-Pak Assay, Chembio Diagnostic Systems, Medford, USA; Simple CHAGAS WB Operon SA, Zaragoza, Spain). Indirect immunofluorescent antibody test.	The patient was considered infected if the results of 2 serology tests were positive. PCR in positive cases.
Roca 2011 Spain	Hospital. Health center in Barcelona	Cross-sectional	To evaluate the prevalence of *T. cruzi* in Latin American adults	2007-2009, 25 months	Immunochromatography assay (ICT) with recombinant *T. cruzi* antigens (TcD, TcE, PEP-2, and SAPA) in whole blood by capillary puncture. Conventional in-house ELISA (cELISA) commercial kit TcD, TcE, PEP-2 y TcLo1.2 (rELISA).	Reactive serum in 2 serology methods: positive. Confirmation: third ELISA (Ortho Clinical Diagnostics) (oELISA).
Rodríguez-Guardado 2009 Spain	Hospital: tropical medicine unit	Prospective	To report the results of a screening program in immigrants from endemic areas	2006-2008, 36 months	ID Chagas (particle gel immunoassay [PaGIA], DiaMed-ID).<br>*T. cruzi* ELISA Ortho, Immunofluorescent antibody test (IFAT) PCR	NR
Salvador 2013 Spain	Hospital: international health program tropical diseases units from the International Health Program of the Catalonian Health Institute, Barcelona (PROSICS)	Prospective	To present clinical and epidemiological data on patients infected by *T. cruzi*.	June 2007-May 2012, 60 months	ELISA with recombinant antigen (BioELISA Chagas, Biokit, Llic de Amunt, Spain)<br>Native ELISA (Ortho T. cruzi ELISA, Johnson & Johnson, High Wycombe, UK or ELISA cruzi, bioMérieux, Marcy-l'Etoile, France).	If results were discordant, Western Blot was requested.
Valerio 2012 Spain	Hospital: 4 centers in the Northern metropolitan area of Barcelona.	Prospective	To report clinical and epidemiological data on patients diagnosed with *T. cruzi* infection	January 2007-December 2011, 48 months	Native ELISA (n-EIA Ortho® T. cruzi Elisa Test System)<br>Recombinant ELISA (r-EIA BioELISA Chagas Biokit®)	NR

NR: not reported or not specified.

APPENDIX IV. STUDIES IN PREGNANT WOMEN UNDERGOING ANTENATAL SCREENING FOR CHAGAS DISEASE

Author, year, country	Setting	Type of study	Objectives	Dates of study, months of follow-up	Screening test: pregnant women	Test in newborn
Ávila Arzanegui 2013 Spain	Hospital antenatal clinics, Gynecology Department of Hospital de Cruces (País Vasco)	Prospective	To determine the prevalence of *T. cruzi* infection, associated epidemiological factors, and the risk of materno-fetal transmission in pregnant women from endemic areas managed in the catchment area	December 2008-January 2010<br>13 months	IFA MarDx®, Inc. Trinity Biotech plc Bray, Co. Wicklow, Ireland, T. cruzi Ab, Dia.Pro, Milan, Italy ORTHO® *T. cruzi* ELISA Test System, Buckinghamshire, United Kingdom<br>High Pure PCR Template Preparation Kit, Roche Diagnostics.	1st week: PCR + serology,<br>1st month: PCR<br>8-9th month: serology.
Barona-Villar 2012 Spain	Gynecology-obstetrics departments of 3 teaching hospitals in Valencia	Cross-sectional	To evaluate the degree of implementation of the early diagnosis protocol for Chagas disease in pregnant women in order to determine the prevalence of infection according to the place of origin and to evaluate the risk of congenital disease.	2009-2010<br>24 months	ORTHO® *T. cruzi* ELISA Test System, Johnson & Johnson, USA, or Chagatek ELISA bioMérieux France and *T. cruzi* Ab, DIAPRO, Diagnostic Bio Probes, Italy. ELISA test (Chagatek ELISA, IDPaGIA Chagas, DiaMed AG, Switzerland, or Stick Chagas, Operon SA, Spain.<br>IF (IFA Kit Trypanosomiasis, MarDx Diagnostics, USA, Innogenetics Ibérica, Spain or Immunofluor Chagas, Biocientífica, Inverness Medical Ibérica, Spain.	Newborn: microhematocrit , IFA , in-house PCR, real-time PCR
Blasco 2011 Spain	Hospital. Screening program for pregnant women at Hospital Universitario La Paz	Retrospective	To analyze the current situation of Chagas disease in terms of pregnancy.	NR	NR	Newborn: microhematocrit and PCR. Check-up at 1 and 9 months.
Martínez 2009 Switzerland	Systematic screening in hospitals in Geneva (Switzerland).	Prospective	To evaluate the prevalence of Chagas disease and the situation of the disease in women from Latin America during delivery, as well as the rate of vertical transmission and follow-up of infected persons.	Year 2008<br>12 months	Serology test: In-house immunofluorescence, Swiss Institute, Basle.	Micro concentration, if PCR negative. If negative, serology at 9 months.
Muñoz(a) 2009 Spain	Gynecology departments of 2 hospitals in Barcelona	Prospective	To determine prevalence of infection by *T. cruzi* in pregnant Latin American women and to evaluate the risk of vertical transmission.	March 2005- September 2007, 31 months	BioELISA Chagas, Biokit S.A. + in-house ELISA Confirmed with ELISA (Ortho-Clinical Diagnosis).	Microhematocrit+PCR
Muñoz-Vilches 2012 Spain	Screening of Latin American women in a hospital in Almeria	Prospective	To present a 4-year experience with screening in pregnant women in Latin America	April 2007- April 2011<br>48 months	In-house ELISA (ELISA-CNMC)+ in-house IFI (IFI-CNM)	NR
Murcia 2013 Spain	Hospital. Follow-up of seropositive pregnant women and their newborns for *T. cruzi.*	Prospective	To analyze the predictive value of the *T. cruzi* PCR assay in pregnant women for the diagnosis of vertical transmission and to evaluate the use of PCR for early detection of infection.	January 2007-December 2011<br>60 months	Test Inmunofluor Chagas, Biocientífica and *T. cruzi* ELISA test system, Ortho Clinical Diagnostics, PCR	Serology and PCR: 0-2, 6, 9, and 12 months of age.
Orti Lucas 2009 Spain	Gynecology, antenatal care, and pediatrics clinics at Hospital Clínico Universitario de Valencia.	Prospective	To determine prevalence in pregnant immigrants from endemic areas with *T. cruzi* infection, to estimate the potential risk of transmission to newborns, and to characterize the epidemiological risk profile of disease-positive women in order to facilitate a diagnostic clinical history in care centers.	February 2005-June 2007<br>28 months	Immunoprecipitation: particle gel immunoassay – DiaMed (IP)<br>Indirect immunofluorescence (IFI) Immunoflour Chagas -Inverness Medical. ELISA Dade Behering	Microhematocrit, serology IgM IFI and PCR (ISCIII Majadahonda)

Author, year, country	Setting	Type of study	Objectives	Dates of study, months of follow-up	Screening test: pregnant women	Test in newborn
Otero 2012 Spain	Screening program in a tertiary hospital in Barcelona	Prospective	To evaluate the 2-year period of the screening program between April 2008 and May 2010, the vertical screening program, coverage, seroprevalence, and rates of vertical transmission.	April 2008–May 2010 25 months	April 2008–November 2009: Novagnost Chagas, Siemens, Marburg, Germany Nov 2009-end: Bioelisa Chagas, Biokit, Barcelona, Spain, and Ortho *T. cruzi* ELISA, Johnson and Johnson New Brunswick, NJ, USA., Western-Blot in-house *T. cruzi* epimastigotes.	0-1 months: Real-time PCR, microhematocrit, if PCR positive, repeat test. Follow-up to 8 months: Serology testing.
Paricio-Talayero 2008 Spain	Maternity departments of 3 regional hospitals in Valencia	Cross-sectional	To determine the prevalence of *T. cruzi* infection in pregnant Latin American women, as well as the risk or cumulative incidence or materno-fetal transmission.	2005 – 2007 27 months	Immunoprecipitation ID-PaGIA Chagas Antibody Test from Diamed-Id and IFI, Innogenetics. PCR, antileishmaniasis test	0-1 months: microhematocrit, PCR. If positive, start treatment. 7 months: immunoprecipitation.
Pérez-Ayala(a) 2011 Spain	Hospital. tropical diseases unit	Prospective	To describe the clinical-epidemiological characteristics of a cohort of Latin American immigrants with chronic *T. cruzi* infection and provide preliminary data on the response to and tolerance of treatment with benznidazole.	2003-2009 72 months	ELISA and IFAT PCR. ECG and Echocardiogram. Esophageal manometry, barium esophagogram	NR
Ramos(a) 2012 Spain	Women attending for materno-fetal health check-ups at a hospital in Elche	Prospective	To investigate the prevalence of *T. cruzi* infection in pregnant immigrants from Central and South America who live in Spain. To investigate transmission of Chagas disease to their newborns.	2006– 2010 60 months	January 2006-March 2008: immunoprecipitation ID PaGIA “Chagas antibody Test”; DiaMed- ID, Barcelona and BioElisa Chagas Biokit SA, Lliça d’Amunt, Barcelona. April 2008-December 2010: Stick Chagas WBs, OPERON S.A. Spain and ELISA Novagnost Chagas IgG Siemens Germany. cELISA, IFI, and real-time PCR.	Serology (rELISA and cELISA January 2006-March 2008 and cELISA and IFI April 2008 to December 2010) and PCR
Ramos 2012 Spain	Hospital	Retrospective	To describe cases of Chagas disease treated in 4 hospitals in the province of Alicante using a similar protocol.	January 2002-May 2011 102 months	ELISA or immunochromatography assay (Novagnost Chagas IgG, NovaTec Immunodiagnostica GmbH, Dietzenbach, Germany, BioElisa Chagas Biokit, Biokit Barcelona, Chagas Stat-Pak Assay, Chembio Diagnostic Systems, Medford, USA Simple CHAGASWB Operon SA, Zaragoza, Spain): IFI	NR
Soriano 2009 Spain	Primary care, pediatrics	Cross-sectional	To measure the prevalence of infection in children and mothers from endemic areas treated in primary care centers and pediatrics departments in Barcelona	March 2002-2007 60 months	Qualitative immunochromatography assay (ICT) (Stat Pak Chagas from Chembio®), capillary puncture, recombinant ELISA (Biokit®) (ELISAr) ELISA *T. cruzi* epimastigote antigens (ELISAc) for confirmation.	No follow-up of newborns in positive cases.

NR, not reported.

APPENDIX V. STUDIES IN IMMIGRANT BLOOD DONORS FROM ENDEMIC AREAS

Author, year, country	Context	Type of study	Objectives	Dates of study, months of follow-up	Screening test	Criteria for positivity
Angheben 2011(d) Italy	3 centers caring for immigrants and a screening program in the population at risk.	Retrospective	To estimate the prevalence of *T. cruzi* infection in Latin American immigrants in Italy and to describe the prevalence of *T. cruzi* infection in patients		Immunochromatography assay (Chagas Quick Test, Cypress Diagnostics, Belgium) and BioELISA Chagas, Biokit S.A., Spain or *Trypanosoma cruzi* lysate, (DRG CHAGAS IgG, Germany).<br><br>Two ELISAs were performed in some cases.	In the case of discordant results, a third test was performed according to the recommendations of the WHO.
Cancino 2015 Spain	Blood bank	Prospective	To investigate parasite load in whole blood and other blood components such as plasma and platelets in donors who are seropositive for *T. cruzi* and to identify differences in parasite levels in blood components.	2011-2013<br>24 months	Bio-ELISA Chagas, Biokit, Grupo Werfen, Barcelona, Spain) *T. cruzi*<br>Recombinant antigen ELISA (Ortho Clinical Diagnostics, Raritan, NJ, USA) with antigen from a whole *T. cruzi* epimastigote extract in the Balearic Islands Blood Bank.	Three positive serology tests.<br>Western blot Ag total *T. cruzi* epimastigote extract Maracay.<br>DNA: PCR (High Pure, Roche, Mannheim, Germany. qPCR (LightCycler 480, Roche)
El Ghouzzi 2010 France	Blood bank in the Blood Transfusion Center “EFS Ile-de-France" in Paris	Prospective	Results of a *T. cruzi* screening program: donors born in Latin America and/or mother born in Latin America and/or return from recent trip (>4-month stay in endemic area).	17 April 2007- 30 October 2008<br>18 months	ELISA cruzi and/or Bioelisa CHAGAS in duplicate. Results repeatedly positive or equivocal: Immunofluor Chagas. Immunofluor Chagas assays *T. cruzi* ELISA, Ortho Clinical Diagnostics (Raritan, NJ, USA). Fourth ELISA Chagatest recombinant ELISA Version 3.0 (Wiener Laboratories, Argentina)	If the 2 ELISAs and IFAs yield positive results, the sample is considered positive.
Gabrielli 2013 Italy	Blood bank	Prospective	To report preliminary results of screening for *T. cruzi* in blood donors born in endemic areas or with mothers from Latin America and Europeans who have lived in or traveled to endemic areas.	2010-2012<br>24 months	Chagas Quick Test, Cypress Diagnostics, Langdorp, Belgium).<br>BioELISA Chagas, Biokit SA, Barcelona, Spain or ELISA NovaLisa Chagas, Nova Tec Immunodiagnostica, GmbH, Dietzenbach, Germany). Giemsa. Genomic DNA (NucleoSpin® tissue, Macherey-Nagel, Düren, Germany) N-PCR.	NR
O'brien 2013 Canada	Blood bank	Retrospective	Results from the first year of selective screening. Report on donor risk factors and result of retrospective research in donors with positive *T. cruzi* antibody titers	17 May 2010- 17 May 2011<br>13 months	Abbott PRISM Chagas (Abbott Laboratories, Wiesbaden, Germany). Immunoblot assay (IB) and PCR Quest Diagnostics (Fairfax, VA) radioimmunoprecipitation assay (RIPA).	Donors were considered to be positive if the result of the IB assay and RIPA were positive.
Pirón 2008 Spain	Blood bank, blood donors in Catalonia	Prospective	To estimate the prevalence of *T. cruzi* infection by means of a test for screening of *T. cruzi* antibodies in donors at risk and persons who have lived in an endemic area for more than 1 year.	September 2005-September 2006<br>12 months	ID-PaGIA, DiaMed, Cressier surMorat, Suiza), Ensayo de Chagas BioELISA (Biokit, Lliçá d'Amunt, España) in -ouse ELISA and ELISA (Ortho-Clinical Diagnostics, Raritan, NJ) and PCR.	Two or more positive results in serology tests.

APPENDIX VI. PREVALENCE OF INFECTION BY *TRYPANOSOMA CRUZI* IN IMMIGRANTS BY COUNTRY OF ORIGIN AND STUDY

**Country of origin[1]**	**Cases**	**%***	**N**	**%***	**N (s)**	**Grouped prevalence immigrants % (95% CI)**	**I^2 %**	**Grouped prevalence blood donors % (95% CI)**	**I^2 %**	**Grouped prevalence pregnant women % (95% CI)**	**I^2 %**	**Global prevalence % (95% CI)**	**I^2 %**	**Heterogeneity between groups**
Total	1,441		19,735		28	13 (7-21)	98.15	0(0-1)	82.06	4(2-7)	96.28	6(3-10)	**98.82**	**0.001**
Bolivia	884	89.66%	3.333	28.05%	25	24 (15-35)	95.36	12(7-18)	0.00	19(13-26)	85	21(15-26)	91.88	0.14
Argentina	32	3.25%	1468	12.35%	15	17 (0-49)	90.20	0 (0-0)	0.00	0 (0-4)	64.93	2(0-5)	82.10	**0.02**
Paraguay	25	2.54%	356	3.00%	15	12 (0-34)	78.76	0 (0-13)	NC	2 (0-7)	0.00	2(0-8)	46.10	0.53
Ecuador	12	1.22%	2152	18.11%	18	0 03(0-1.51)	34.65	0 (0-0.16)	0.00	0.07 (0-0.61)	15.16	0(0-0.08)	2.99	0.65
Brazil	8	0.81%	982	8.26%	16	0 0(0-1.15)	51.14	0 (0-0))	0.00	0 02(0-2.80)	52.04	0(0-0)	44.26	0.07
Colombia	7	0.71%	1499	12.61%	15	0 (0-1.28)	15.56	0 (0-0)	0.00	0 23(0-1.69)	59.18%	0(0-0.17)	36.92	0.31
Peru	6	0.61%	723	6.08%	16	0.52 (0-3.86)	60.37	0 (0-0.71)	NC	0 (0-1.69)	0.00	0(0-0.53)	22.67	0.23
Honduras	5	0.51%	134	1.13%	10	2 (0-14)	NC	0 (0-12)	NC	0(0-3)	0.00	0(0-2)	0.00	0.29
Chile	3	0.30%	239	2.01%	13	2 (0-42)	58.19	0 (0-0)	0.00	1 (0-9)	0.00	0(0-0)	27.90	0.17
Venezuela	2	0.20%	362	3.05%	14	0 (0-7)	21.60	0 (0-1)	0.00	0 (0-1)	0.00	0(0-0)	0.00	0.29
Nicaragua	1	0.10%	52	0.44%	8	0 (0-4)	NC	0 (0-14)	NC	1 (0-24)	NC	0(0-1)	0.00	0.42
Mexico	1	0.10%	159	1.34%	13	1 (0-23)	0.001	0 (0-0))	NC	0 (0-6)	0.00	0(0-0)	0.00	0.05
Costa Rica	0	0.00%	9	0.08%	3	0 (0-79)	NC	0 (0-21)	NC	NC	NC	0(0-18)	0.00	0.70
Panama	0	0.00%	18	0.15%	9	0 (0-79)	NC	0 (0-43)	NC	0 (0-13)	0.00	0(0-9)	0.00	0.93
Uruguay	0	0.00%	216	1.82%	11	0 (0-4)	0.00	0 (0-0)	0.00	0 (0-0)	0.00	0(0-0)	0.00	NC
Guatemala	0	0.00%	21	0.18%	6	0 (0-79)	NC	0 (0-3)	NC	0 (0-39)	NC	0(0-3)	0.00	0.69
Guyana	0	0.00%	161	1.35%	1	NC	NC	NC	NC	NC	NC	NC	NC	NC

[1]Ordered by number of cases. N, total population screened. *Percentage with respect to the total number of cases and persons screened whose origin was known (n=986).
The total includes studies in which the patients' origin is not detailed by country. N(s), number of studies. I^2, heterogeneity as a percentage. NC, not calculated (owing to an insufficient number of cases to perform the meta-analysis or to calculate the heterogeneity value). The meta-analysis was performed using the metaprop command with the Freeman-Tukey double arcsine transformation to include studies with no cases detected. The model used was the random effects model. In the case of studies that included persons from nonendemic countries or results from patients aged <14 years, the results were recalculated by country and as totals (Angheban, Avila-Arzan, Frank, Paricio Talayero)

APPENDIX VII. PREVALENCE OF *TRYPANOSOMA CRUZI* INFECTION IN PREGNANT WOMEN AND RATE OF VERTICAL TRANSMISSION

Author Year	Mean (SD) age of pregnant women, years	Origin of women screened	Prevalence of *T. cruzi*	Origin of cases	Mean (SD) age of pregnant women, years	Symptoms and/or organ involvement	Rate of vertical transmission	Symptoms of neonatal infection
Ávila- Arzanegui 2013	28.5 (5.3)	Bolivia 45.6%, Colombia 13.3%, Paraguay 13.3%, Ecuador 10.1%, Peru 7%, Brazil 4.4%, Venezuela 2.5%, Argentina 1.3%, Uruguay 0.6%, Mexico 0.6%, Cuba 0.6%, Dominican Republic 0.6%	12.17% (19/156)*	Bolivia 84.2% Paraguay 2 and Brazil 1. In Bolivian women: Santa Cruz 13/17, Cochabamba 1, Chuquisaca 2, and Tarija 1.	28.4 (4.9)	Eight with symptoms and no cardiac, digestive, or organ involvement	5.26% 1/17	Asymptomatic, term pregnancy without complications. PCR + at first month of life, confirmed at month 2.
Barona-Vilar 2012	29 (6) range 14–48	Ecuador 30%, Bolivia 21.7%, Colombia 17.8%, Argentina 6.4%	11.44% (226/1975)	Bolivia 94.7%	NR	NR	3.54% 8/226 Bolivia 100%	Hepatosplenomegaly 1/8
Blasco 2011	NA	Bolivia 100%	100%	Bolivia 100%	32 range 25-40	NR	0% 0/5	NA
Martínez 2009	30.4 (5.7)	Brazil 39%, Bolivia 22.9%, Peru 9.2%, and other 28.9%.	1.97% (6/305)	Bolivia 100%	NR	NR	25% 2/8 Bolivia 100%	Prematurity 100%
Muñoz(a) 2009	31 range 18-43	Ecuador 34%, Peru 16%, Bolivia 14%, and Colombia 12%.	3.41% (46/1350)	Bolivia 91%	NR	NR	7.32% 3/41	Asymptomatic
Muñoz-Vilches 2012	NR	Ecuador 25.3%, Argentina 21.5%, Colombia 17.2%, Peru 16.9%, Bolivia 6.1%, Brazil 5.7%, Venezuela 2.7%, Chile 2.3%, Mexico 0.8%, Uruguay, Paraguay, Panama, Honduras (1.6% each).	1.53% (4/261)	Argentina 1, Peru 1, and Bolivia 2	NR	1 case of grade I esophageal involvement	0% 0/4	NA
Murcia 2013	NA	Case follow-up	100%	Bolivia 96.6% y Paraguay 3.4%	29.1 (6.5) range 15-43	NR	13.85% 9/65 Bolivia 100%	3/9 1st twin: LBW, abdominal distension, hepatosplenomegaly, jaundice, myocarditis, heart failure, difficulty breathing. 2nd twin: LBW and splenomegaly. 3rd case Apgar 1'-5' (3-6), LBW, abdominal distension, hepatosplenomegaly, heart failure, severe pulmonary hypertension, and

Author Year	Mean (SD) age of pregnant women, years	Origin of women screened	Prevalence of *T. cruzi*	Origin of cases	Mean (SD) age of pregnant women, years	Symptoms and/or organ involvement	Rate of vertical transmission	Symptoms of neonatal infection
								difficulty breathing. Outcome favorable.
Orti-Lucas 2009	25.9 (5)	Ecuador 30.8%, Bolivia 20.1%, Colombia 16.4%, and Argentina 13.1%	9.66% (37/383)	Bolivia 54.2%, Argentina 13.5%, Colombia 8.1%, Brazil 5.4%, Ecuador 5.4%<br>Other (1 case each, 13.5%).	24.3	Symptoms*:<br>Cardiological: 40%<br>Digestive: 31.6%	2.70%<br>1/37 Bolivia 100%	Asymptomatic. Infant's results positive at 8 months
Otero 2012	29.5 (6)	Bolivia 22.90%	3.48% (22/633)	Bolivia 95.5% and Ecuador 4.5%	NR	Cardio: 1 (LVD + nodal dysfunction)<br>Digestive: 4 (3 dolichosigmoid and 1 dolichocolon)	5%<br>1/20	1/1 LBW, prematurity (35 weeks), hepatosplenomegaly, cholestasis, and cytolysis.
Paricio-Talayero 2008	28.3 (5.8)<br>range: 14 -45	Ecuador 31.2%, Colombia 20.9%, Bolivia 22% Argentina 10% Uruguay 4.1%, Venezuela 3%, Brazil 3% Peru 1.4%, and other 4%	4.65% (29/624)	Bolivia 82.8%, Colombia 17%, and Ecuador 14%	NR	Asymptomatic	0%<br>0/29	NA
Pérez-Ayala 2011	NR	Not detailed (Latin American women)	NA	NR	NR	NR	0%<br>0/11	NA
Ramos(a) 2012	Median 28.9, range 16-45<br>Age < 26 years. 32.5%	Colombia 30.1%, Ecuador 23.9%, Argentina 14.7%, Paraguay 8.4%, and Bolivia 7.2%.	1.28%<br>(7/545)	Bolivia 57%, Paraguay 43%	NR	Asymptomatic	0%<br>0/7	NA
Ramos 2012	NA	Latin America	100%	NR	NR	NR	6.25%<br>2/32	Asymptomatic

NA, not applicable; NR, not reported; LVD, left ventricular dysfunction; LBW, low birth weight (according to the study definition); SD, standard deviation.
*Percentage of the total who presented symptoms irrespective of severity or without specifying the symptoms.

APPENDIX VIII. PREVALENCE OF *T. CRUZI* INFECTION IN BLOOD DONORS

Author Year	Population screened, sex, mean (SD), age	Origin of population screened	Prevalence, origin of cases	Cases: age, sex, symptoms
Angheben 2011(d)	28<br>Men 50%<br>Mean age 39<br>range 21–55	Brazil 28.5%, Peru 17.9%, Chile 14.3%, Colombia 10.7%, Venezuela 10.7%	0% (95% CI, 0%-12.06%)<br>0/28	NR
Cancino 2015	1,201	Argentina 54%, Ecuador 30%, Bolivia 8% Venezuela 7%	1.9%<br>23/1201:<br>Argentina 0.61%, Ecuador 0.27%, Bolivia 16.03%, Venezuela 1.19%	NR
El Ghouzzi 2010	972	Latin America 100%<br>Central America 13%<br>South America 87%	0.3%<br>3/972:<br>Latin America 0.31%<br>Central America 1.61%, El Salvador 14.29%<br>South America 0.12% Bolivia 12.5%	Women 2<br>Age range 26-43
Gabrielli 2013	102<br>Men 58.26%<br>Age 37.5<br>range 19-66	Peru 28%, Ecuador 14%, Argentina 14%, Bolivia 5%<br><br>Italy (26): history of travel to endemic areas.	1.0%<br>1/102	Age < 40 years, asymptomatic
O'brien 2013	7,255		0.2%<br>13/7,255<br>Paraguay 9, Argentina 2, other 4 with mothers or grandmothers born in Russia. The mothers of the 2 patients born in Canada were from South America.	NR
Pirón 2008	1524<br>Men 51%<br>Age 35 (11)	Colombia 22.3% Argentina 19.5% Ecuador 14.6%	0.7%<br>10/1524<br>Bolivia: 10.2%<br>Bolivia (6, Santa Cruz 4 and Cochabamba 2), Argentina (2), Ecuador (1), and Paraguay (1)	Women 60%<br>Mean age 38.5 years

APPENDIX IX. PREVALENCE OF INFECTION BY *T. CRUZI* AND CHARACTERIZATION OF CASES AMONG IMMIGRANTS IN GENERAL

Author Year	Total population screened, sex, age	Origin of the population screened	Prevalence, origin of cases	Cases, age, sex	Symptoms and/or cardiac involvement	Symptoms and/or gastrointestinal involvement
Angheben 2011(i)	266<br>Women 65%<br>Mean age 34, range 4- 83 years.	Brazil 30%, Bolivia 28.2%, Peru 9.8%, Colombia 9.7%, Argentina 5.3%, México 3.8%, Ecuador 3.5%	30/266: 11.3%<br>Bolivia 23, Argentina 2, Paraguay 2, Brazil 1, Ecuador 1, México 1	NR	NR	NR
Favila 2015	251<br>Men: 57.8%<br>Mean (SD) age: 34.62 (9.3) years	Bolivia 100%	48/251: 19.1%.<br>By province<br>La Paz 6.4%, Santa Cruz 19.6%, Cochabamba 19.8%, Chuquisaca-Potosí-Tarija 68.2%. Other provinces with a lower risk 8.3%	Women 54.2%<br>Mean (SD) age:<br>35.5 (10.2) years	NR	NR
Frank1997	100<br>Men 60%. Median age: 28 years (range 20-55 years)	Peru 44%, Bolivia 21%, Ecuador 16%, Colombia 12%<br>Other countries 7%	2/100: 2.0%. 1 Bolivia, 1 Peru	1 man and 1 woman aged 45 and 35 year, respectively	NR	NR
Jackson 2010	1,012<br>Women 83%<br>Mean age 37.2 (SD) 11.3<br>96% without a residence permit.	Bolivia 48%, Brazil 25%, Colombia 6%, Peru 6%, Ecuador 5%, Paraguay 3%, Nicaragua 2%, Honduras 2%, and other countries 3%	130/1012: 12.8%<br>127 Bolivia, 2 Argentina, and 1 Brazil. The last 3 had lived in Bolivia for some time	NR	14/124 (11.3%) ECG abnormalities. Classification according to Brazilian Consensus[2].<br>12 (9.7%) grade A, 1 grade B2 (0.8%), and other 12 (9.7%) with normal ECG had symptoms. 4/7 with echocardiographic signs, low grade ventricular dysfunction, and 1 dilated coronary sinus. Rhythm abnormalities in a further 2 cases (Holter).	21 patients (16.9%) reported dysphagia (n=10) and/or severe constipation (n=16). Barium meal performed in 16 patients, grade I esophageal involvement in 1 patient (0.8%). 109 patients with indeterminate chronic infection.
Lescure 2008	NR	NR	9 French Guiana (1), 8 Bolivia: Santa Cruz and Cochabamba	Woman from French Guiana (26 years), 8 remaining patients: median age, 38; range (24-48). 4 men	8 bradycardia, 1 case of atypical acute precordial pain.<br>2 patients needed a pacemaker owing to AV block.	No digestive involvement reported.
Lescure 2009	254<br>Women 59.8%, Median age 33 years (11-63)	Bolivia 87.4%, Colombia 4.7%, Ecuador 2.4%	60/254: 23,6%<br>Bolivia: 87.4%, La Paz, Cochabamba, Sucre, Santa Cruz	NR	18/60: cardiac symptoms: precordial pain, dyspnea, palpitations, malaise.	17/60 with functional digestive signs: constipation, abdominal pain, gastroesophageal reflux, regurgitation
Muñoz 2009	489	Latin America: Bolivia 55%, Ecuador 21%, Colombia 5%, Peru 4% Argentina 4% Brazil 3%, Honduras	202/489: 41.3%	NR	[1]30/159 (19%) with at least 1 ECG abnormality, systolic dysfunction in 7 /30, 1 in heart transplant program, 4	34 with constipation and 3 with dysphagia, digestive involvement: 15/159 (9%) 11 patients with dolichocolon.

Author Year	Total population screened, sex, age	Origin of the population screened	Prevalence, origin of cases	Cases, age, sex	Symptoms and/or cardiac involvement	Symptoms and/or gastrointestinal involvement
		3%, Paraguay 1.2%, other countries 4%			with diastolic dysfunction, 2 required a pacemaker. 1 case of bradycardia and asymptomatic bifascicular block died suddenly.	Diameter of colon > 6 cm in a further 3 patients. 1 patient with grade I achalasia.
Navarro 2011	276<br>Women 64.9%<br>Median age 32<br>Range (1–68) years	Bolivia 76.4%, Ecuador 10.5%, Peru 5.8%, other countries 7.2%	44/276: 15,9%<br>100% Bolivia, 33 from Cochabamba and Santa Cruz. Rate of seroprevalence among Bolivians, 20.9%	30 women of reproductive age, 11 of whom were pregnant	NR	NR
Pérez Molina 2011	1145	121 reported by Madrid and 3 by Switzerland.	126/1145: 11.0%<br>Bolivia 96.0%, Argentina 1.6%, Paraguay 1.6%, Ecuador ,0.8%. More common: Cochabamba 32.3% and Santa Cruz 29.8%.	Women: 65.3%<br>Age, median (IQR)<br>35 (29–45) years	NR	NR
Pérez-Ayala(a) 2011	1146		357/1146: 31.2%<br>Bolivia 97%	241<br>Women 67.5%<br>Median age, 36 (IQR 29-44) years	Heart involvement only in 17.1%, 3 with dilated cardiomyopathy (1 had received a transplant and 2 on waiting list),<br>2 pacemakers (complete AV block), 12 cardiomyopathy, and 29 ECGs suggestive of Chagas disease.	13/ 252 (3,5%)<br>1 megasyndrome (colon and sigmoid)<br>3 with abnormalities in esophageal manometry,<br>4 dolichocolon and megacolon, and 5 dolichosigmoid.<br>Cardiodigestive involvement, 1.6%
Ramos(b) 2012	201<br>Women 56.7%<br>Median age: 30 years	Paraguay 128 and Bolivia 73	13/201: 6,5%<br>Bolivia 7, Paraguay 6.<br>In Bolivians, 9.59% (95% CI, 4.72%–18.5%). In Paraguayans, 4.69% (95% CI, 2.17%–9.85%)	6/7 Bolivia (Cochabamba and Santa Cruz<br>PCR positive 61.5%	Palpitations 7, precordial pain 5	5 constipation
Ramos 2012			128<br>Bolivia 78.9%, Paraguay 8.6%, Argentina 5.5%, Colombia 2.3%, Brazil 1.6%, and Ecuador 1.6%.	Median age: 35 years<br>Range 0-72 years<br>Women 63.3%.<br>Mean stay Spain 3.9 years, range 15 days to 11.1 years	55 asymptomatic patients<br>Palpitations 40%, chest pain 26.4%, dyspnea 15.5%. Syncope 10%, associated with cardiomyopathy (OR=6.5; 95% CI, 1.5–27.1), edema 2.7%, cardiomegaly 7%, ECG abnormalities 23.1%.<br>Cardiac involvement 25%, pacemaker 1, heart transplant 1, on waiting list 1. ECG abnormalities and/or ECC 20.	Constipation 19.1%<br>Gastroesophageal reflux 12.2%.<br>Dysphagia 5.5%, Abnormal esophagogram findings 7.7%.<br>Gastrointestinal involvement, 1.9%<br>Normal findings in barium enema, 100%
Roca 2011	766<br>Men 39,8% Mean (SD) age 36.43 (12.2) years	Peru 22.9%, Ecuador 22.3%, Bolivia 16.6%, Colombia 13.3%, Argentina 8.2%, Venezuela 4%, Brazil 3%, Chile 2.9%, Paraguay 2.5%, Uruguay 1.8%, Honduras 1.6%, and other countries 0.9%	22/766: 2.9%<br>Bolivia :21, Paraguay: 1.	NR	2/21<br>9.5%<br>1: right bundle branch block, 1 inverted T wave, 2 cardiodigestive.	2 cardiac involvement<br>1 right bundle branch block with left anterior hemiblock and hypotonic lower esophageal sphincter. 1 right bundle branch block and severely hypotonic lower esophageal sphincter and distal esophageal hypoperistalsis

Author Year	Total population screened, sex, age	Origin of the population screened	Prevalence, origin of cases	Cases, age, sex	Symptoms and/or cardiac involvement	Symptoms and/or gastrointestinal involvement
Rodríguez-Guardado 2009	64<br>Women 73%<br>Mean age 36 range 28-48 years	Ecuador 39%, Brazil and Bolivia 15.6% each, Colombia 14%, Dominican Republic 9.6%, and Cuba and Paraguay (3.1% each).	9/64: 14,1%,<br>Bolivia (Santa Cruz) 6, Paraguay 2, and Brazil 1.<br>In Bolivians: 60%.	4 women and 5 men, mean age 45 years, mean time of residence 1,335 days.	Two reported palpitations	NR
Salvador 2013	NA	NA	1274<br>Bolivia 97%, Argentina 1.3%, Ecuador 0.5%, Paraguay 0.4%, other countries 11(0.8%).	Women: 67.5%<br>Mean (range) age: 37.7 (18–81)	Cardiac involvement 16.9%<br>Symptoms (n: 1274) Chest pain 6.5%, Dyspnea 5.2%, palpitations 5.1%, syncope 1.1%, ACV 0.2%, w/heart symptoms 13.8%.<br>Kuschnir classification (n: 1125) **0**: 83.1%, **I**: 15%, **II**: 0.9% y **III** 1%, 8 third-degree block requiring pacemaker.	Symptoms: constipation 21.8%, dysphagia 3.2%, dyspepsia 3.1%, gastroesophageal reflux disease 1.7%, with digestive symptoms 26.1%.<br>Digestive involvement 14.8% ,104 dolichocolon, 31 megacolon, 25 with esophageal abnormalities
Valerio 2012	NA	NA	139, Bolivia 94.2% Ecuador (3), Argentina (2), Brazil (1), Uruguay (1), Colombia (1).	Mean (SD) age 37.79 (9.9) Women 61.2%	44.6% Staging according to modified Brazilian consensus[2] on Chagas cardiomyopathy: 0 (60.4%), a (28.7%), b1 (2.9%), b2 (7.2%), c (0.7%), d (0)	Abnormal esophagography findings 18:13 gastroesophageal reflux disease, 4 megasyndrome (esophagus), 1 megasyndrome (stomach). Abnormal esophageal manometry 23: 10 mild-to-moderate motor dysfunction, 4 severe motor dysfunction, 6 hypotonic lower esophageal sphincter, 1 partial aperistalsis, 2 pseudoachalasia.<br>Abnormal barium enema findings 3: 2 dilated descending colon, 1 megacolon

[1]Criteria of Gascón et al for suspicion of cardiac involvement (Muñoz 2009). ECG criteria: bradycardia, AV block, any type of hemiblock, tachyarrhythmia, Q waves, premature ventricular contractions, T waves. Clinical criteria: dyspnea, orthopnea, and signs of congestive heart failure, syncope, dizziness, palpitations, and precordial pain suggestive of heart disease (Gascón et al. 2007)

[2]Brazilian Consensus: 0 ECG normal, (a) (abnormal ECG, normal Echo, no signs of congestive heart failure, (b1) abnormal ECG, abnormal Echo, EF>45%, no signs of heart failure, (b2) Abnormal ECG, abnormal Echo, EF<45%, no signs of heart failure, (c) Abnormal ECG, abnormal Echo, presence of compensated heart failure), (d) Abnormal ECG, abnormal Echo, refractory congestive heart failure.

APPENDIX X. TREATMENT OF CHAGAS DISEASE IN IMMIGRANTS IN GENERAL.

Author year[1]	Cases of *T. cruzi* infection	Trypanocidal agent	Started treatment	Completed treatment	Cases with adverse effects	Adverse effects (description)	Additional data
Lescure 2008	9	Benznidazole, 1 patient switched to nifurtimox.	9/9<br>100.0%	6<br>66.7%	5/9<br>55.6%	DRESS (1), peripheral neuropathy (4), 1 patient switched to nifurtimox and completed treatment appropriately.	One acute case and 8 chronic cases
Pérez-Ayala(a) 2011	357	Benznidazole 5 mg/kg in 2 or 3 doses divided daily for 60 days. Three patients received nifurtimox as second-line treatment. Well-tolerated in all cases.	195/252<br>77.4%	104/195<br>53.30%	77/148 52.0%	77/148 had adverse events<br>Cutaneous toxicity: 68.7%<br>Gastrointestinal symptoms: 20%<br>Nervous system disorders 16.2%.<br>Mild leukopenia: 6.5%<br>Taste disorders: 2.6%.	Treatment discontinued in 29.7%, although the adverse effects were mild.
Ramos 2012	128	5 mg/kg/d of benznidazole (100 mg) for 45-60 days.	76/108<br>70.4%	57<br>75.0%	35/76<br>46.1%	35/76 had adverse events.	Treatment interrupted in 17.1% because of adverse events. Six interrupted treatment for other reasons.
Rodríguez-Guardado 2009	9	Benznidazole 5–7 mg/kg/d for 60 days	9/9<br>100.0%	9<br>100.0%	0/9<br>0.0%	No signs of hepatic or liver toxicity, good tolerance, PCR turned negative at 3 months of treatment.	NR
Salvador 2013	1274	Benznidazole in 629 (98.9%) nifurtimox 1.1%.	636/1245<br>51.1%	523<br>82.2%	462/636<br>72.6%	At least 1 adverse event 72.6%.<br>Cutaneous toxicity 53%<br>Gastrointestinal events 20.6%<br>Increased transaminases 11.5%<br>Neurological disorders 10% (peripheral neuropathy and dysgeusia)<br>Reduced neutrophils 3.5%<br>General adverse effects 29.1% (headache, somnolence, weakness, and bone and joint pain).	Treatment was stopped owing to toxicity in 13.7%.<br><br>Incomplete treatment in 4.1% for reasons not associated with adverse events.
Valerio 2012	139	Benznidazole 116 (82.1%), nifurtimox 1 patient	116/139<br>83.5%	89<br>76.7%	56/110<br>50.9%	Of the 110 patients monitored, 56 (50.9%) had drug-related adverse effects, and treatment was suspended in 21 (19.1%)<br>Headache 8.2% Gastrointestinal effects 6.4%<br>Dermatologic effects 34.5%<br>Peripheral neuropathy 3.6%, Arthritis 2.7%, Fever 0.9%	1 patient switched to nifurtimox (10 mg/kg/24 h for 60 days) and completed treatment. NR

[1]Studies in which treatment received is reported. NR, not reported.

APPENDIX XI. TREATMENT OF CHAGAS DISEASE IN NEWBORNS OF CHAGASIC MOTHERS.

Author Year	Cases of *T. cruzi* in pregnant women	Infected newborns receiving treatment<br>Treatment received	Trypanocidal drug	Women who started treatment after pregnancy	Completed treatment	Cases with adverse effects	Adverse effects	Additional data
Ávila Arzanegui 2013	19	1/1<br>Benznidazole (10 mg/kg/d for 60 days), which the patient tolerated without adverse reactions and with negative PCR at 1 month and negative serology at 6 months after completing treatment.	Benznidazole	6/9<br>66.7%	6<br>100.0%	NR	NR	NR
Barona-Vilar 2012	226	8/8 benznidazole (7 mg/kg/d for 60 days) without adverse events	Benznidazole	NR	NR	NR	NR	NR
Martínez 2009	6	2/2<br>Nifurtimox with no complications	Nifurtimox (2)<br>Benznidazole (1)	3/3<br>100.0%	1<br>33.3%	1/3<br>33.3%	Intolerance (1)	Only 3 mothers contacted.
Muñoz(a) 2009	46	3/3<br>Benznidazole 5 mg/kg/d for 60 days good tolerance.	NR	NR	NR	NR	NR	NR
Muñoz-Vilches 2012	4	NR	Benznidazole	2/4<br>50.0%	NR	NR	NR	NR
Murcia 2013	59	9/9<br>Benznidazole: no adverse events with treatment.	Benznidazole	NR	NR	NR	NR	No vertical transmission in 10/59 pregnant women who had previously received treatment
Otero 2012	22	1/1<br>Empirical treatment with benznidazole. At 25 days of treatment, negative PCR; at 7 months, negative serology NR	NR	NR	NR	NR	NR	NR
Ramos(a) 2012	7	NR	Benznidazole (7.5 mg/kg/d for 60 days)	5/7<br>71.4%	4<br>80.0%	2/5<br>40.0%	2 skin rash	Of the 2 patients who presented with skin rash, 1 interrupted treatment.
Soriano 2009	5	NR	Benznidazole	5/5<br>100.0%	NR	NR	Headache, urticaria, and anorexia during the first 2-3 weeks	NR

NR, not reported.

APPENDIX XI. TREATMENT OF CHAGAS DISEASE (trials)

STUDY	POPULATION	TREATMENTS	OUTCOMES	EFFICACY
**STOP-CHAGAS Trial**<br>(NCT01377480)	120 subjects from Latin America and Spain<br>Asymptomatic Chagas carriers	Posaconazole 400 mg<br>Benznidazole 200 mg + placebo 10 mg<br>Benznidazole 200 mg + posaconazole 400 mg<br>Placebo 10 mg<br>all treatments administered twice a day	**Primary**<br>Proportion of subjects with persistent negative RT-PCR by day 180<br>**Secondary**<br>Negative RT-PCR at 360 days.	**Primary outcome**<br>13.3% posaconazole monotherapy; 10% placebo<br>80% benznidazole + posaconazole<br>86.7% benznidazole monotherapy<br>($p < 0.0001$ vs. posaconazole/placebo)<br>**High RT-PCR conversion 30 days**<br>93.3% posaconazole monotherapy; 10% placebo<br>88.9% benznidazole + posaconazole<br>89.7% benznidazole monotherapy<br>($p < 0.0001$ vs. posaconazole/placebo)<br>**High RT-PCR conversion 60 days**<br>90% posaconazole monotherapy; 16.7% placebo<br>92.3% benznidazole + posaconazole<br>89.3% benznidazole monotherapy<br>($p < 0.0001$ vs. posaconazole/placebo)<br>**High RT-PCR conversion 360 days**<br>16% posaconazole monotherapy; 17% placebo<br>96% benznidazole + posaconazole<br>96% benznidazole monotherapy<br>($p < 0.0001$ vs. posaconazole/placebo)<br>Permanent **discontinuation** was reported in 19 patients (31.7%).<br>**SAFETY** Serious adverse events were rare (6 patients) in the benznidazole-treated patients.
**E1224 Study Group trial**<br>(NCT01489228)	231 adults with confirmed diagnosis of *Trypanosoma cruzi* infection from 2 outpatient units in Bolivia	High-dose E1224 (8 weeks, total dose 4000 mg)<br>Low-dose E1224 (8 weeks, 2000 mg)<br>Short-dose E1224 (4 weeks + 4 weeks placebo, 2400 mg)<br>Benznidazole (60 days, 5 mg/kg per day)<br>Placebo (8 weeks)	**Primary**<br>Parasitological response to E1224<br>**Secondary**<br>Parasitological response to benznidazole<br>Sustained parasitological response until 1 year<br>Parasite clearance<br>changes in parasite load<br>Conversion to negative response<br>Changes in levels of biomarkers<br>Complete response	During the treatment phase E1224 showed **parasite clearance**, but it was not sustained (with low-dose and short-dose regimens)<br>**Sustained response**: 29% (95% CI, 16.4-44.3) in the high-dose regimen vs 82% (95% CI, 67.9-92.0) in benznidazole vs 9% (95% CI, 2.4-20.4) in placebo ($p<0.0001$).<br>Parasite levels in the low-dose and short-dose E1224 groups gradually returned to placebo levels.<br>**SAFETY** 81% participants developed treatment-emergent adverse events (3% experienced serious adverse events).

5. REFERENCES

METHODOLOGY

Higgins JPT, Thompson SG, Deeks JJ, Altman DG. Measuring inconsistency in meta-analyses. British Medical Journal 2003; 327: 557-560.

Higgins JPT, Green S (editors). Cochrane Handbook for Systematic Reviews of Interventions Version 5.1.0 [updated March 2011]. The Cochrane Collaboration, 2011. Available from www.handbook.cochrane.org

Hoy D, Brooks P, Woolf A, Blyth F, March L, Bain C, et al. Assessing risk of bias in prevalence studies: modification of an existing tool and evidence of interrater agreement. J Clin Epidemiol. 2012;65(9):934-9.

Moher D1, Liberati A, Tetzlaff J, Altman DG; PRISMA Group.Preferred reporting items for systematic reviews and meta-analyses: the PRISMA statement. PLoS Med. 2009;6 (7):e1000097.

Nyaga VN, Arbyn M, Aerts M. Metaprop : a Stata command to perform meta-analysis of binomial data. Arch Public Health.2014; 72:1–10.

Tricco AC, Antony J, Zarin W, Strifler L, Ghassemi M, Ivory J, Perrier L, Hutton B, Moher D, Straus SE. A scoping review of rapid review methods. BMC Med. 2015;13 : 224.

STUDIES EVALUATED FOR DRAFTING OF THE CLINICAL QUESTION

Angheben A, Anselmi M, Gobbi F, Marocco S, Monteiro G, Buonfrate D, et al. Chagas disease in Italy: breaking an epidemiological silence. Euro Surveill. 2011;16(37).

Avila Arzanegui O, Liendo Arenaza P, Martinez Indart L, Martinez Astorkiza T, Pocheville Guruceta MI, Egurbide Arberas MV. [Prevalence of Trypanosoma cruzi infection and vertical transmission in Latin-American pregnant women in a health area of Biscay]. Enferm Infecc Microbiol Clin. 2013;31(4):210-6.

Barona-Vilar C, Gimenez-Marti MJ, Fraile T, Gonzalez-Steinbauer C, Parada C, Gil-Brusola A, et al. Prevalence of Trypanosoma cruzi infection in pregnant Latin American women and congenital transmission rate in a non-endemic area: the experience of the Valencian Health Programme (Spain). Epidemiol Infect. 2012;140(10):1896-903.

Blasco G L, Nuñez M V, Cruceyra B M, Magdaleno D F, García B S. Enfermedad de Chagas y embarazo. Revista chilena de obstetricia y ginecología. 2011;76:162-8.

Cancino-Faure B, Fisa R, Riera C, Bula I, Girona-Llobera E, Jimenez-Marco T. Evidence of meaningful levels of Trypanosoma cruzi in platelet concentrates from seropositive blood donors. Transfusion. 2015;55(6):1249-55.

Crespillo-Andújar C, Venanzi-Rullo E, López-Vélez R, Monge-Maillo B, Norman F, López-Polín A, Pérez-Molina JA. Safety Profile of Benznidazole in the Treatment of Chronic Chagas Disease: Experience of a Referral Centre and Systematic Literature Review with Meta-Analysis. Drug Saf. 2018;41(11):1035-1048.

El Ghouzzi MH, Boiret E, Wind F, Brochard C, Fittere S, Paris L, et al. Testing blood donors for Chagas disease in the Paris area, France: first results after 18 months of screening. Transfusion. 2010;50(3):575-83.

Favila Escobio P, Ribas J, M GM, Rodriguez-Ramirez G, Vicens-Ferrer J, Esteva M. Prevalence of Chagas disease in the Bolivian population of Majorca (Spain). Gac Sanit. 2015;29(4):288-91.

Frank M, Hegenscheid B, Janitschke K, Weinke T. Prevalence and epidemiological significance of Trypanosoma cruzi infection among Latin American immigrants in Berlin, Germany. Infection. 1997;25(6):355-8.

Gabrielli S, Girelli G, Vaia F, Santonicola M, Fakeri A, Cancrini G. Surveillance of Chagas disease among at-risk blood donors in Italy: preliminary results from Umberto I Polyclinic in Rome. Blood Transfus. 2013;11(4):558-62.

Jackson Y, Getaz L, Wolff H, Holst M, Mauris A, Tardin A, et al. Prevalence, clinical staging and risk for blood-borne transmission of Chagas disease among Latin American migrants in Geneva, Switzerland. PLoS Negl Trop Dis. 2010;4(2):e592.

Lescure FX, Canestri A, Melliez H, Jaureguiberry S, Develoux M, Dorent R, et al. Chagas disease, France. Emerg Infect Dis. 2008;14(4):644-6.

Lescure FX, Paris L, Elghouzzi MH, Le Loup G, Develoux M, Touafek F, et al. [Experience of targeted screening of Chagas disease in Ile-de-France]. Bull Soc Pathol Exot. 2009;102(5):295-9.

Martinez de Tejada B, Jackson Y, Paccolat C, Irion O. [Congenital Chagas disease in Geneva: diagnostic and clinical aspects]. Rev Med Suisse. 2009;5(222):2091-2, 4-6.

Morillo CA, Marin-Neto JA, Avezum A, Sosa-Estani S, Rassi A Jr, Rosas F, Villena E, Quiroz R, Bonilla R, Britto C, Guhl F, Velazquez E, Bonilla L, Meeks B, Rao-Melacini P, Pogue J, Mattos A, Lazdins J, Rassi A, Connolly SJ, Yusuf S; BENEFIT Investigators. Randomized Trial of Benznidazole for Chronic Chagas' Cardiomyopathy. N Engl J Med. 2015;373(14):1295-306.

Morillo CA, Waskin H, Sosa-Estani S, Del Carmen Bangher M, Cuneo C, Milesi R, Mallagray M, Apt W, Beloscar J, Gascon J, Molina I, Echeverria LE, Colombo H, Perez-Molina JA, Wyss F, Meeks B, Bonilla LR, Gao P, Wei B, McCarthy M, Yusuf S; STOP-CHAGAS Investigators. Benznidazole and Posaconazole in Eliminating Parasites in Asymptomatic T. Cruzi Carriers: The STOP-CHAGAS Trial. J Am Coll Cardiol. 2017;69(8):939-947.

Muñoz J, Coll O, Juncosa T, Verges M, del Pino M, Fumado V, et al. Prevalence and vertical transmission of Trypanosoma cruzi infection among pregnant Latin American women attending 2 maternity clinics in Barcelona, Spain. Clin Infect Dis. 2009;48(12):1736-40.

Muñoz J, Gomez i Prat J, Gallego M, Gimeno F, Trevino B, Lopez-Chejade P, et al. Clinical profile of Trypanosoma cruzi infection in a non-endemic setting: immigration and Chagas disease in Barcelona (Spain). Acta Trop. 2009;111(1):51-5.

Muñoz-Vilches MJ, Salas J, Cabezas T, Metz D, Vásquez J, Soriano MJ. Cribado de Chagas en mujeres gestantes latinoamericanas. Experiencia en el Poniente Almeriense. Enfermedades Infecciosas y Microbiología Clínica. 2012;30(7):380-2.

Murcia L, Carrilero B, Munoz-Davila MJ, Thomas MC, Lopez MC, Segovia M. Risk factors and primary prevention of congenital Chagas disease in a nonendemic country. Clin Infect Dis. 2013;56(4):496-502.

Navarro M, Perez-Ayala A, Guionnet A, Perez-Molina JA, Navaza B, Estevez L, et al. Targeted screening and health education for Chagas disease tailored to at-risk migrants in Spain, 2007 to 2010. Euro Surveill. 2011;16(38).

O'Brien SF, Scalia V, Goldman M, Fan W, Yi QL, Dines IR, et al. Selective testing for Trypanosoma cruzi: the first year after implementation at Canadian Blood Services. Transfusion. 2013;53(8):1706-13.

Ortí Lucas RM, Parada Barba MC. Prevalencia de tripanomiasis americana en mujeres gestantes de un área de salud: Valencia, 2005-2007. Revista Española de Salud Pública. 2009;83:543-55.

Otero S, Sulleiro E, Molina I, Espiau M, Suy A, Martin-Nalda A, et al. Congenital transmission of Trypanosoma cruzi in non-endemic areas: evaluation of a screening program in a tertiary care hospital in Barcelona, Spain. Am J Trop Med Hyg. 2012;87(5):832-6.

Paricio-Talayero JM, Benlloch-Muncharaz MJ, Collar-del-Castillo JI, Rubio-Soriano A, Serrat-Perez C, Magraner-Egea J, et al. [Epidemiological surveillance of vertically-transmitted Chagas disease at three maternity hospitals in the Valencian Community]. Enferm Infecc Microbiol Clin. 2008;26(10):609-13.

Perez-Ayala A, Perez-Molina JA, Norman F, Navarro M, Monge-Maillo B, Diaz-Menendez M, et al. Chagas disease in Latin American migrants: a Spanish challenge. Clin Microbiol Infect. 2011;17(7):1108-13.

Perez-Molina JA, Perez-Ayala A, Parola P, Jackson Y, Odolini S, Lopez-Velez R, et al. EuroTravNet: imported Chagas disease in nine European countries, 2008 to 2009. Euro Surveill. 2011;16(37).

Pirón M, Verges M, Munoz J, Casamitjana N, Sanz S, Maymo RM, et al. Seroprevalence of Trypanosoma cruzi infection in at-risk blood donors in Catalonia (Spain). Transfusion. 2008;48(9):1862-8.

Ramos JM, Torrus D, Amador C, Jover F, Perez-Chacon F, Ponce Y, et al. Multicenter epidemiological and clinical study on imported Chagas diseases in Alicante, Spain. Pathog Glob Health. 2012;106(6):340-5.

[Ramos(a)2012] Ramos JM, Milla A, Rodríguez JC, Lopez-Chejade P, Flores M, Rodriguez JM, et al. Chagas disease in Latin American pregnant immigrants: experience in a non-endemic country. Arch Gynecol Obstet. 2012;285(4):919-23.

[Ramos(b)2012] Ramos JM, Ponce Y, Gallegos I, Flores-Chavez M, Canavate C, Gutierrez F. Trypanosoma cruzi infection in Elche (Spain): comparison of the seroprevalence in immigrants from Paraguay and Bolivia. Pathog Glob Health. 2012;106(2):102-6

Roca C, Pinazo MJ, Lopez-Chejade P, Bayo J, Posada E, Lopez-Solana J, et al. Chagas disease among the Latin American adult population attending in a primary care center in Barcelona, Spain. PLoS Negl Trop Dis. 2011;5(4):e1135.

Rodriguez-Guardado A, Rodriguez M, Alonso P, Seco C, Flores-Chavez M, Mejuto P, et al. Serological screening of Chagas disease in an immigrant population in Asturias, Spain proceeding from Chagas-endemic areas. Scand J Infect Dis. 2009;41(10):774-6.

Salvador F, Trevino B, Sulleiro E, Pou D, Sanchez-Montalva A, Cabezos J, et al. Trypanosoma cruzi infection in a non-endemic country: epidemiological and clinical profile. Clin Microbiol Infect. 2014;20(7):706-12.

Soriano Arandes A, Munoz Gutierrez J, Verges Navarro M, Castells Domenech C, Portus Vinyeta M, Gascon Brustenga J. Prevalence of Chagas disease in the Latin American immigrant population in a primary health centre in Barcelona (Spain). Acta Trop. 2009;112(2):228-30.

Torrico F, Gascon J, Ortiz L, Alonso-Vega C, Pinazo MJ, Schijman A, Almeida IC, Alves F, Strub-Wourgaft N, Ribeiro I; E1224 Study Group. Treatment of adult chronic indeterminate Chagas disease with benznidazole and three E1224 dosing regimens: a proof-of-concept, randomised, placebo-controlled trial. Lancet Infect Dis. 2018;18(4):419-430.

Valerio-Sallent L, Roure S, Basile L, Ballesteros LA, Sabria M, Rodrigo C, et al. [A clinical and epidemiological study of the Trypanosoma cruzi infected population in the north metropolitan area of Barcelona]. Rev Clin Esp. 2012;212(7):329-36.

ADDITIONAL REFERENCES

D'Albuquerque LA, Gonzalez AM, Filho HL, Copstein JL, Larrea FI, Mansero JM, et al. Liver transplantation from deceased donors serologically positive for Chagas disease. Am J Transplant. 2007;7(3):680-4.

De Paula EV, Goncales NS, Xueref S, Addas-Carvalho M, Gilli SC, Angerami RN, et al. Prevalence of transfusion-transmitted Chagas Disease among multitransfused patients in Brazil. BMC Infect Dis. 2008;8:5.

Emődy L. Chagas disease, update on a migration related tropical disease in Europe. Boletin Salud Pública, Organización Mundial de la Salud Issue 2 Junio 2014.

Enfermedad de Chagas y donación de sangre. Grupo de trabajo Donación de Sangre e Inmigracion. Dirección General de Salud Pública y Sanidad Exterior. Ministerio de Sanidad y Politica Social. Madrid 2009.

European Centre for Disease Prevention and Control. Assessing the burden of key infectious diseases affecting migrant populations in the EU/EEA. Stockholm: ECDC; 2014.

Flores-Chavez M, Fernandez B, Puente S, Torres P, Rodriguez M, Monedero C, et al. Transfusional chagas disease: parasitological and serological monitoring of an infected recipient and blood donor. Clin Infect Dis. 2008;46(5):e44-7.

Fores R, Sanjuan I, Portero F, Ruiz E, Regidor C, Lopez-Velez R, et al. Chagas disease in a recipient of cord blood transplantation. Bone Marrow Transplant. 2007;39(2):127-8.

Howard EJ, Xiong X, Carlier Y, Sosa-Estani S, Buekens P. Frequency of the congenital transmission of Trypanosoma cruzi: a systematic review and meta-analysis. BJOG. 2014;121(1):22-33.

Imaz Iglesia, I., García San Miguel, L., Blasco Hernández, T., Martín Águeda, B., González-Enríquez, J., Ayala Morilas, L. E., García Pérez, L., Sarría Santamera, A. «Evaluación económica de distintas estrategias de cribado de la Enfermedad de Chagas en España». IPE 2015/72. Agencia de Evaluación de Tecnologías Sanitarias (AETS) - Instituto de Salud Carlos III, Madrid. 2015

Martin-Davila P, Fortun J, Lopez-Velez R, Norman F, Montes de Oca M, Zamarron P, et al. Transmission of tropical and geographically restricted infections during solid-organ transplantation. Clin Microbiol Rev. 2008;21(1):60-96.

Organización Mundial de la Salud. La Enfermedad de Chagas (tripanosomiasis americana)
Nota descriptiva N°340,Marzo de 2016.
http://www.who.int/mediacentre/factsheets/fs340/en/

Perez-Molina JA, Perez-Ayala A, Moreno S, Fernandez-Gonzalez MC, Zamora J, Lopez-Velez R. Use of benznidazole to treat chronic Chagas' disease: a systematic review with a meta-analysis. The Journal of antimicrobial chemotherapy. 2009;64(6):1139-47

Requena-Mendez A, Aldasoro E, de Lazzari E, Sicuri E, Brown M, Moore DA, et al. Prevalence of Chagas disease in Latin-American migrants living in Europe: a systematic review and meta-analysis. PLoS Negl Trop Dis. 2015;9(2):e0003540

World Health Organization (WHO) (2015) World Health Organization (WHO) Chagas disease in Latin America: an epidemiological update based on 2010 estimates. Weekly Epidemiological Record. 2015;90:33–44.

Villalba R, Fornes G, Alvarez MA, Roman J, Rubio V, Fernandez M, et al. Acute Chagas' disease in a recipient of a bone marrow transplant in Spain: case report. Clin Infect Dis. 1992;14(2):594-5

STUDIES NOT EVALUATED FOR DRAFTING OF THE CLINICAL QUESTION

Abstracts and communications to scientific meetings:

Alegria I, Coll MT, Bachs MR, Botet F, Anquela I, Laya M, et al. Congenital Chagas disease: the importance of screening before the delivery. Our experience in a regional hospital in Catalonia. Trop Med Int Health 2011;16:370.

Bassa N, Soldevila F, Soriano P, Gassiot P, Mora C. Prevalence of Chagas infection in pregnant women native of South America in an area of northeast Spain. Clin Chem Lab Med 2011;49:S516.

Cruz CC, Piris BS, Caman o Gutierrez I. Screening for Chagas disease: a priority in our health system. J Matern Fetal Neonatal Med 2010;23:391.

Jackson Y, Delieutraz-Marchand J, Reis C, Benchouk M, Mauris A, Sizonenko S, et al. Prevalence of Chagas disease among pregnant Latin American women and their newborns in Geneva, Switzerland. Trop Med Int Health 2009;14:238.

Strohmeyer M, Gabrielli S, Bartalesi F, Mantella A, Aiello KH, Di Tommaso MR, et al. Screening of congenital Chagas disease in Florence, Italy. Trop Med Int Health 2009;14:239.

Flores-Chavez M, Merino F, Garcia-Bujalance S, Martin-Rabadan P, Merino P, Garcia-Bermejo I, et al. Surveillance of Chagas disease in pregnant women in Madrid (Spain), 2008–2010. Trop Med Int Health 2011;16:368.

Merino P, Gonzalez F, Amor E, Gastanaga T, Urbon J, Picazo J. Seroprevalence of Chagas' Disease in pregnant woman from Latin America. Screening and follow up of their children in a non-endemic country. Trop Med Int Health 2009;14:238.

Salas J, Cabezas T, Munoz MJ, Vazquez J, Martin R, Benitez C, et al. Chagas screening in pregnant women from Latin America: experience in western Almeria. Trop Med Int Health 2011;16:288

Sombrero HR, Arnal IO, Carrasco EG. DO we need the screening of congenital Chagas disease in our country. J Matern Fetal Neonatal Med 2010;23:160–1.

Studies with indirect data: registers

Herrador Z, Rivas E, Gherasim A, Gomez-Barroso D, Garcia J, Benito A, et al. Using hospital discharge database to characterize Chagas disease evolution in Spain: there is a need for a systematic approach towards disease detection and control. PLoS Negl Trop Dis. 2015;9(4):e0003710.

Case reports

Centers for Disease Control and Prevention. Congenital Transmission of Chagas Disease — Virginia, 2010. MMWR 2012;61:477-478

Review articles

Basile L, Jansa JM, Carlier Y, Salamanca DD, Angheben A, Bartoloni A, et al. Chagas disease in European countries: the challenge of a surveillance system. Euro Surveill. 2011;16(37).

Basile L, Oliveira I, Ciruela P, Plasencia A, Working Group For Developing The Catalonian Screening Programme For Congenital Transmission Of Chagas D. The current screening programme for congenital transmission of Chagas disease in Catalonia, Spain. Euro Surveill. 2011;16(38).

Carlier Y. Globalization of Chagas disease (American trypanosomiasis): the situation in Europe and Belgium. Bull Mem Acad R Med Belg. 2011;166(10-12):347-55; discussion 56-7.

de Ory Manchon F. [Seroepidemiological surveys of non vaccine-preventable diseases and their interest in public health]. Rev Esp Salud Publica. 2009;83(5):645-57.

Garraud O, Andreu G, Elghouzzi MH, Laperche S, Lefrere JJ. Measures to prevent transfusion-associated protozoal infections in non-endemic countries. Travel Med Infect Dis. 2007;5(2):110-2.

Gascon J, Bern C, Pinazo MJ. Chagas disease in Spain, the United States and other non-endemic countries. Acta Trop. 2010;115(1-2):22-7.

Navarro M, Perez-Ayala A, Guionnet A, Perez-Molina JA, Navaza B, Estevez L, et al. Targeted screening and health education for Chagas disease tailored to at-risk migrants in Spain, 2007 to 2010. Euro Surveill. 2011;16(38).

Perez-Molina JA, Norman F, Lopez-Velez R. Chagas disease in non-endemic countries: epidemiology, clinical presentation and treatment. Curr Infect Dis Rep. 2012;14(3):263-74.

Pinazo MJ, Gascon J. The importance of the multidisciplinary approach to deal with the new epidemiological scenario of Chagas disease (global health). Acta Trop. 2015;151:16-20.

Roca Saumell C, Soriano-Arandes A, Solsona Diaz L, Gascon Brustenga J, Grupo de consenso Chagas APS. [Consensus document for the detection and management of Chagas disease in primary health care in a non-endemic areas]. Aten Primaria. 2015;47(5):308-17.

Rodriguez-Morales AJ, Benitez JA, Tellez I, Franco-Paredes C. Chagas disease screening among Latin American immigrants in non-endemic settings. Travel Med Infect Dis. 2008;6(3):162-3.

Soriano-Arandes A, Angheben A, Serre-Delcor N, Trevino-Maruri B, Gomez IPJ, Jackson Y. Control and management of congenital Chagas disease in Europe and other non-endemic countries: current policies and practices. Trop Med Int Health. 2016;21(5):590-6.

Studies performed in endemic countries

Bua J, Volta BJ, Velazquez EB, Ruiz AM, Rissio AM, Cardoni RL. Vertical transmission of Trypanosoma cruzi infection: quantification of parasite burden in mothers and their children by parasite DNA amplification. Trans R Soc Trop Med Hyg. 2012;106(10):623-8.

Moscatelli G, Berenstein A, Tarlovsky A, Siniawski S, Biancardi M, Ballering G, et al. Urban Chagas disease in children and women in primary care centres in Buenos Aires, Argentina. Mem Inst Oswaldo Cruz. 2015;110(5):644-8.

Fabbro DL, Danesi E, Olivera V, Codebo MO, Denner S, Heredia C, et al. Trypanocide treatment of women infected with Trypanosoma cruzi and its effect on preventing congenital Chagas. PLoS Negl Trop Dis. 2014;8(11):e3312.

Salas Clavijo NA, Postigo JR, Schneider D, Santalla JA, Brutus L, Chippaux JP. Prevalence of Chagas disease in pregnant women and incidence of congenital transmission in Santa Cruz de la Sierra, Bolivia. Acta Trop. 2012;124(1):87-91.

Cost studies

Imaz-Iglesia I, Miguel LG, Ayala-Morillas LE, Garcia-Perez L, Gonzalez-Enriquez J, Blasco-Hernandez T, et al. Economic evaluation of Chagas disease screening in Spain. Acta Trop. 2015;148:77-88.

Sicuri E, Bardaji A, Nhampossa T, Maixenchs M, Nhacolo A, Nhalungo D, et al. Cost-effectiveness of intermittent preventive treatment of malaria in pregnancy in southern Mozambique. PLoS One. 2010;5(10):e13407.

Studies on blood/blood product donation

Kitchen AD, Hewitt PE, Chiodini PL. The early implementation of Trypanosoma cruzi antibody screening of donors and donations within England: preempting a problem. Transfusion. 2012;52(9):1931-9.

Duplicate studies

Perez-Ayala A, Perez-Molina JA, Norman F, Monge-Maillo B, Faro MV, Lopez-Velez R. Gastro-intestinal Chagas disease in migrants to Spain: prevalence and methods for early diagnosis. Ann Trop Med Parasitol. 2011;105(1):25-9.

Ramos JM, Milla A, Sanchez V, Verges M, Toro C, Gutierrez F. [Prenatal screening for Trypanosoma cruzi and human T lymphotropic virus types 1 and 2 in pregnant Latin American women]. Enferm Infecc Microbiol Clin. 2009;27(3):165-7.
